# Biopotential of Underutilized *Rosaceae* Inflorescences: LC-DAD-MS Phytochemical Profiles Associated with Antioxidant, Antidiabetic, Anti-Inflammatory and Antiproliferative Activity *In Vitro*

**DOI:** 10.3390/plants11030271

**Published:** 2022-01-20

**Authors:** Ivana Šola, Danijela Poljuha, Maja Mikulic-Petkovsek, Dino Davosir, Marija Pinterić, Josipa Bilić, Robert Veberic, Metka Hudina, Gordana Rusak

**Affiliations:** 1 Department of Biology, Faculty of Science, University of Zagreb, Horvatovac 102a, 10000 Zagreb, Croatia; ddavosir@stud.biol.pmf.hr (D.D.); gordana.rusak@biol.pmf.hr (G.R.); 2 Department of Agriculture and Nutrition, Institute of Agriculture and Tourism, Karla Huguesa 8, 52440 Poreč, Croatia; danijela@iptpo.hr; 3 Department of Agronomy, Biotechnical Faculty, University of Ljubljana, Jamnikarjeva 101, 1000 Ljubljana, Slovenia; maja.mikulic-petkovsek@bf.uni-lj.si (M.M.-P.); robert.veberic@bf.uni-lj.si (R.V.); metka.hudina@bf.uni-lj.si (M.H.); 4 Division of Molecular Medicine, Ruđer Bošković Institute, 10000 Zagreb, Croatia; Marija.Pinteric@irb.hr; 5 METRIS Research Centre, Istrian University of Applied Sciences, Riva 6, 52100 Pula, Croatia; jbilic@iv.hr

**Keywords:** 5-lipoxygenase, α-amylase, α-glucosidase, *Chaenomeles*, colorectal carcinoma, hepatocellular carcinoma, keratinocytes, *Malus*, metabolomics, *Prunus*

## Abstract

The aim of this work was to assess the biopotential of the young inflorescence tissues of *Prunus*, *Malus* and *Chaenomeles* in order to evaluate the possibility of their application in the food industry, and to provide a polyphenolic fingerprint for their quality control. The contents of different bioactive compounds and their antioxidant capacities were spectrophotometrically measured, the main phenolic compounds were identified and quantified using LC-DAD-MS, the antidiabetic potential was determined using α-amylase and α-glucosidase inhibition assays, the anti-inflammatory potential was determined using a 5-lipoxygenase inhibition assay, and the cytotoxicity was determined by MTT assay. Using one-way ANOVA, principal component analysis, hierarchical clustering and Pearson’s correlation coefficient, the relations between the samples, and between the samples and the measured parameters, were revealed. In total, 77 compounds were identified. The concentration of sugars was low in *M. purpurea,* at 1.56 ± 0.08 mg/g DW. The most effective sample in the inhibition of antidiabetic enzymes and anti-inflammatory 5-lipoxygenase was *C. japonica*. The inhibition of α-glucosidase was strongly positively correlated with the total and condensed tannins, procyanidin dimers and procyanidin tetramer, and was very strongly correlated with chlorogenic acid. In α-amylase inhibition, *C. japonica* and *P. serrulata* ‘Kiku Shidare Zakura’ were equally efficient to the standard inhibitor, maltose. The most effective in the growth and proliferation inhibition of HepG2, HCT116 and HaCaT cells was *P. avium*. The results suggest *Prunus*, *Malus* and *Chaenomeles* inflorescences as functional food ingredients.

## 1. Introduction

Inflorescence, as a precursor of a fruit, is composed of several types of metabolically very active tissues. In recent years, the biopotential of the bioactive compounds of this plant structure has been recognized. Namely, the significant bioactivities of inflorescences of industrial hemp [1], *Musa* species [2], *Sorbus aucuparia* [3], *Cistus salviifolius* [4], *Lonicera japonica* [5], and *Caryota urens* [6] have been revealed. Moreover, the flower buds of *C. salviifolius* have been shown to have a higher concentration of phenolics, better antioxidant and anti-superoxide dismutase activity, and stronger cytotoxic activity against human breast cancer and ovarian cancer cells than leaves [4]. Recently, the intrinsic antiradical activity of industrial hemp’s inflorescences’ water extracts and mechanisms related to their anti-inflammatory, antiproliferative and antimycotic activity have been revealed [1]. The inflorescences of *Musa* species are one of the most widely consumed vegetables in the Southeast Asian region, and their biological activities are well known.

The use of inflorescences of *Rosaceae* varieties, except those of *Sorbus* spp. [3], is not that common. *Sorbus* spp. inflorescences have a higher concentration of total phenolics than the commonly consumed fruits [3]. Furthermore, the inflorescences of different S*orbus* species have higher antioxidant activity than their leaves or fruits [7]. Moreover, the extremely high content of phenolics in *S. aucuparia* inflorescences suggests their great potential as a source for natural health products [3,7]. Different organs of *Prunus*, *Malus*, and *Chaenomeles* taxa have already been investigated for their phytochemical, nutraceutical and bioactivity potential [8,9,10,11,12,13]. However, the inflorescences have been neglected so far.

Therefore, the aim of this work was to assess the biopotential of the young inflorescence tissues of selected *Prunus*, *Malus*, and *Chaenomeles* using a combination of spectrometric, chromatographic, cell culture and chemometric analyses, as well as to evaluate the possibility of their application in the food industry. For that purpose, we (a) spectrophotometrically measured the content of different types of bioactive compounds (total phenolics, flavonoids, non-flavonoids, total and condensed tannins, and soluble sugars), and their antioxidant capacity by the three methods (ABTS, FRAP and DPPH); (b) identified and quantified the main phenolic compounds using the LC-DAD-MS method; (c) determined the antidiabetic potential of the inflorescences using α-amylase and α-glucosidase inhibition assays; (d) measured the anti-inflammatory potential using a 5-lipoxygenase inhibition assay; (e) assessed the cytotoxicity toward HepG2, HCT 116 and HaCaT cells by MTT assay; and (f) statistically—using one-way ANOVA, principal component analysis, hierarchical clustering and Pearson’s correlation coefficient—revealed the relations between the samples, as well as between the samples and the measured parameters. This study is the first to present a comprehensive LC-DAD-MS polyphenolic profile of the inflorescences of *Prunus avium* (L.) L. (*Pa)*, *Prunus serrulata* Lindl. (*Ps)*, *Prunus serrulata* ‘Kiku Shidare Zakura’ *(PsKss)*, *Malus* x *purpurea* (E.Barbier) Ehder (*Mp)*, *Malus floribunda* Siebold ex Van Houtte (*Mf)* and *Chaenomeles* *japonica* (Thunb.) Lindl. ex Spach (*Cj)*, providing a fingerprint for the future quality control of these biomaterials, and the first report on their bioactivity.

## 2. Results and Discussion

The food industry mainly focuses on *Rosaceae* fruits, and somewhat less on the flowers, stems and leaves [8], which are mostly used in their dried form for tea. Because inflorescences are composed of several types of metabolically very active tissues, it can be assumed that these plant reproductive structures contain a significant concentration of bioactive phytochemicals, and that they present thus-far-underutilized plant potential. Therefore, in this study, for the first time, we offer data on phytochemical profile and bioactivity of inflorescences of *Pa*, *Ps*, *PsKss*, *Py*, *Mp*, *Mf* and *Cj*.

### 2.1. Spectrophotometric Analysis of the Phytochemical Content and Antioxidant Capacity of the Inflorescences

All of the the tested samples had a similar or higher amount of total phenolics than the commonly used flower buds of *Capparis spinosa* [14]. The highest amount of total phenolics (53.12 ± 0.79 mg GAE/g DW), flavonoids (38.89 ± 4.04 mg CE/g DW) and nonflavonoids (31.32 ± 0.71 mg GAE/g DW) were recorded in *PsKss* (Table 1). Because only about 10% of the medicinal plant material has total phenolics in a concentration higher than 5% DW of GAE [7,15], *PsKss* inflorescence is among the plant materials with the highest concentration of these compounds. Compared to the concentration of the total flavonoids in many other commonly used medicinal plants [15], the tested inflorescences had a similar or higher concentration, suggesting their potential for the food industry. Moreover, very recently, it was shown that inflorescences of *Pa* have far more total phenolics and flavonoids than stems and kernels [16].

In order to reduce the global rise of overweightness and obesity, the content of sugars, as a source of calories, in food should be diminished. As expected, the level of sugars in the inflorescences was lower than that in the more often-used fruits. The interval of the concentrations in the inflorescences was between 1.56 ± 0.08 mg/g DW in *Mp* and 8.61 ± 0.12 mg/g DW in *Py* (Table 1), which is relatively low compared to the concentrations recorded in *M. domestica* fruits [17] and *Pa* fruits [10]. Namely, the ripe flesh and skin of cultivated stone and pome fruits usually contain soluble sugars in the amount of 70–90% of the DW [18]. This is one of the main reasons why *Rosaceae* inflorescences have a high potential in the food industry, and should be considered a potent biomaterial. 

The antioxidant potential was assessed using three standard methods (ABTS, FRAP and DPPH), and each of them revealed *PsKss* as the most potent sample (Table 1). Because this taxon had significantly more total phenolics, flavonoids, nonflavonoids (Table 1), and total identified hydroxycinnamic acids than the other samples (Table 2), with the predominant ones being caffeic acid hexoside 1,3-caffeoylquinic acid, 4-caffeoylquinic acid, 5-caffeoylquinic acid 1 (chlorogenic acid), and *p*-coumaric acid hexoside 1 and 2, we assume that these compounds significantly contributed to its antioxidant potential. The same relations between the content of phenolic compounds and antioxidant activity between different parts of *Pa* have already been revealed as well [16,19]. The antioxidant activity of *Cj* inflorescences was similar to that of its fruits [20]. However, inflorescences of Pa showed higher antioxidant potential than the stems and kernels [16]. *Mf* had a higher antioxidant capacity than *Mp* (Table 1), and this is probably related to the higher amount of identified hydroxycinnamic acids, especially chlorogenic acid, flavanols, flavanones, flavonols and procyanidins (Table 2). Both *Mf* and *Mp* dry inflorescences showed higher antioxidant activity than the fresh flowers of different *Malus* cultivars [21], highlighting their potential for use in the food industry.

### 2.2. LC-DAD-MS Analysis of the Individual Phenolics in the Inflorescences

Detailed studies on the phytochemical profile of *Rosaceae* plant parts other than the fruits (roots, stems, leaves, and flowers) are very scarce. The identification and quantification of chemical compounds in the inflorescences of *Rosaceae* contributes to the understanding of the basis of their bioactivity. Just recently, the first results of the phytochemical analyses of the stems, leaves and flowers of *Pa* were published by Jesus et al. [8]; however, inflorescences were not included. Our study is the first to present a comprehensive LC-DAD-MS polyphenolic profile of selected *Rosaceae* inflorescences, and it provides a fingerprint for the future quality control of the selected inflorescences. 

We identified, in total, 77 phenolic compounds (Table 2, Supplementary Table S1). The representative chromatograms, at different wavelengths, of each of the species are presented in Supplementary Figure S1; the highest number of individual compounds (46), as well as the highest concentration of total identified compounds (82.77 ± 0.26 mg/g DW), were recorded in *Mf* (Table 2; Supplementary Figure S2). This species also contained the highest concentration of procyanidins, with 35.73 ± 0.66 mg/g DW (Table 1). On the other hand, *Pa* had the lowest concentration of total identified compounds, 18.05 ± 2.34 mg/g DW. In the inflorescences of this species, we identified 36 compounds, which is significantly more than were identified in its stems, leaves and fruits [8,9,19,22,23], emphasising the wealth of different compounds in inflorescences. However, this was the only sample in which we did not identify individual procyanidins (Table 2). The most individual compounds represented with the highest concentration among the samples were detected in *Py* (20), representing 49% of all of the identified compounds in this species (Table 2, Supplementary Figure S2). 

The individual phenolic with the highest concentration in *Pa* was di-caffeoylquinic acid (3.12 ± 0.08 mg/g DW), in *Ps* it was quercetin-acetyl hexoside (4.00 ± 0.40 mg/g DW), in *PsKss* it was caffeic acid hexoside (15.48 ± 2.23 mg/g DW), in *Py* and *Mf* it was procyanidin trimer (11.05 ± 0.30 mg/g DW and 8.66 ± 1.08 mg/g DW, respectively), in *Mp* it was phloridzin (5.14 ± 0.74 mg/g DW), and in *Cj* the most represented phenolic was procyanidin dimer (7.90 ± 1.48 mg/g DW) (Table 2). In each of the *Prunus* inflorescence samples, the concentration of quercetin-3-rutinoside was present with a higher concentration than that of quercetin-3-galactoside, which is opposite to the inflorescence of *P. serotina* [24], and *P. avium* inflorescence had a similar concentration of chlorogenic acid to the flowers of *P. padus* [25].

In *Pa*, *PsKss* and *Py*, the predominant identified compounds were hydroxycinnamic acids in *Ps* and *Mp* flavonols, and in *Mf* and *Cj* they were procyanidins (Table 1). In *Pa*, epicatechin was the main flavanol, while catechin was present in a smaller concentration, and the same ratio had already been recorded for fruits as well [23]. Among hydroxybenzoic acids, we identified gallic acid in the concentration of 0.28 ± 0.06 mg/g DW. This was previously found in *Pa* stems and fruits as well; however, it was not found in leaves and flowers [8]. 

*Mf* had a significantly higher concentration of total identified flavonols and procyanidins than other samples, at 21.60 ± 0.16 mg/g DW and 35.73 ± 0.66 mg/g DW, respectively (Table 1). It had far more chlorogenic acid than its whole fruit, flesh or peel [26]. Chalcones were identified in *Mf* and *Mp* only, in total concentrations of 6.80 ± 0.24 mg/g DW and 5.61 ± 0.28 mg/g DW, respectively (Table 1). Among them, phloridzin predominated, with 23 ± 0.52 mg/g DW and 5.14 ± 0.74 mg/g DW, respectively. Phloretin xylosylglucoside and trilobatin were represented in inverse proportions in *Mf* and *Mp*; in *Mf* trilobatin predominated with a five-times-higher concentration than in *Mp*, while in *Mp* phloretin xylosylglucoside predominated with a two-times-higher concentration than in *Mf* (Table 1). This suggests that the ratio of phloretin xylosylglucoside and trilobatin could be used as a phytochemical differentiator between these two species. 

In *Cj,* we identified 43 polyphenolic compounds (Table 2), compared to the 20 compounds identified in the fruits [27]. This emphasises the value of inflorescences in the richness and variety of polyphenolic compounds. The representation of flavanols, flavonols and flavanones in *Cj* inflorescences was similar to that in *Chaenomeles maulei* fruit juices [28]. The most important polyphenol group was procyanidins (Table 1 and Table 2), which was similar to its leaves [12] and fruits [27].

### 2.3. Antidiabetic Activity of the Inflorescences

The antidiabetic activity of the samples was assessed via the inhibition of α-amylase and α-glucosidase, which are enzymes required for carbohydrate digestion. These enzymes are targets not only when attempting to alleviate diabetes but also hyperlipidemia, obesity and caries [29]. The potential of *Pa* flower, stem and leaf extracts in the inhibition of α-glucosidase has been recognised [8]. Stem extract inhibited α-glucosidase significantly more than extracts of *P. avium Saco* and *Hedelfinger* fruits [22], which shows the biopotential of *Rosaceae* plant parts other than the commonly consumed fruits.

**Figure 1:**
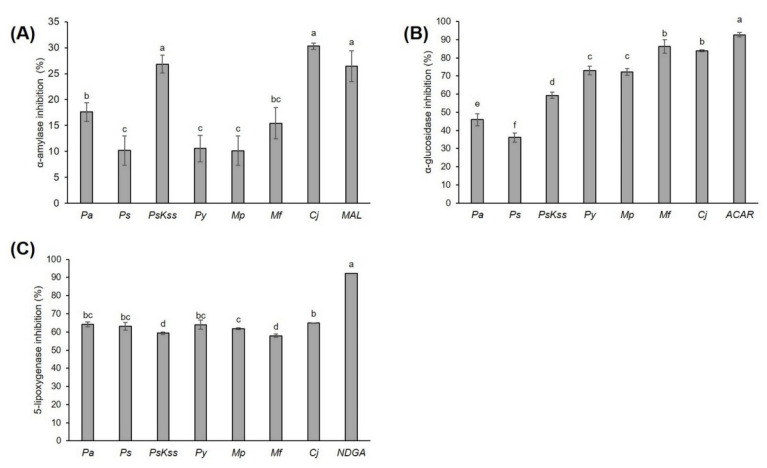
Inhibition of (**A**) α-amylase, (**B**) α-glucosidase, and (**C**) 5-lipoxygenase activity by *Rosaceae* inflorescences’ extracts (0.80 mg/mL, 0.55 mg/mL and 1.45 mg/mL, respectively). The values represent the mean ± standard deviation of three replicates. Different letters indicate a significant difference among the values (ANOVA, Duncan test, *p* ≤ 0.05). *Pa* = *P. avium*, *Ps* = *P. serrulata*, *PsKss* = *P. serrulata* ‘Kiku Shidare Zakura’, *Py* = *P. yedoensis*, *Mp* = *M. purpurea*, *Mf* = *M. floribunda*, *Cj* = *Chaenomeles japonica*, MAL = maltose 0.80 mg/mL, ACAR = acarbose 0.55 mg/mL, NDGA = nordihydroguaiaretic acid 0.15 mg/mL.

This work is the first report on α-amylase and α-glucosidase inhibition by *Rosaceae* inflorescences. Each of the tested samples more efficiently inhibited α-glucosidase than α-amylase (Figure 1A,B).

The same tendency was recorded with extracts of *Chaenomeles* fruits as well [20]. However, the fact that the inhibitions of α-amylase, *Cj* and *PsKss* were equally efficient to the standard maltose at the same concentration is very interesting and promising. Moreover, both samples even showed a tendency to be more effective than maltose. This emphasizes the high antidiabetic potential of the mentioned inflorescences, and we suggest further *in vivo* investigations of the antidiabetic activity of these biomaterials and the mechanisms behind this activity. One of the possible intermediates in this α-amylase inhibitory activity could be phenolic compounds. Indeed, phenolic acids and flavonoids bind covalently to α-amylase, forming quinones or lactones that react with nucleophilic groups of the enzyme, thus altering the enzyme’s activity. *Cj* had the highest concentration of total condensed tannins, 51.68 ± 0.38 mg CE/g DW, while *PsKss* had the highest concentration of total phenolics, flavonoids and nonflavonoid compounds, at 53.12 ± 0.79 mg GAE/g DW, 38.89 ± 4.04 mg CE/g DW and 31.32 ± 0.71 mg GAE/g DW, respectively (Table 1). Therefore, we assume that, among those measured, these compounds mostly contributed to the inhibition of α-amylase. Moreover, α-amylase showed the highest positive Pearson’s correlation coefficient in its total condensed tannins and total flavonoids, with r = 0.585 and r = 0.458, respectively (Table 3). 

As in the case of the total condensed tannins, as determined by the spectrophotometric method, which were most represented in the *Cj* (Table 1), the LC-DAD-MS method also revealed the highest concentrations of individual procyanidins in this species (Table 2). Compared to the other samples, *Cj* had significantly more procyanidin dimer 1, 2, 4, 5, 6 and procyanidin tetramer, and also 5-*p*-coumaroylquinic acid 2, naringenin hexoside, isorhamnetin acetyl hexoside 1 and 2, myricetin rutinoside and syringetin acetyl hexoside 2 (Table 2), so we hypothesyze that among these compounds one might potentially find new strong inhibitor/s of α-amylase. Here, we would especially emphasise procyanidins, as they were present in higher amounts—procyanidin dimer 1 even being present at 7.90 ± 1.48 mg/g DW—than the other compounds, and probably mainly contributed to the inhibition of this enzyme. As a support, the significance of tannins for α-amylase inhibition was recognised in work with sorghum as well [30]. An extract of pinhão coat (*Araucaria angustifolia*) rich in condensed tannins also effectively inhibited α-amylase [29]. On the other hand, in *PsKss* extract, the predominant compound was caffeic acid hexoside 1 with 15.48 ± 2.23 mg/g DW, which is more than 37% of all of the identified compounds in this species, and this might be one of the key contributors to the inhibition of α-amylase activity. Moreover, recently, fruits from the genus *Prunus* have been suggested for the preparation of extracts with antidiabetic activities [31]. 

**Figure 2:**
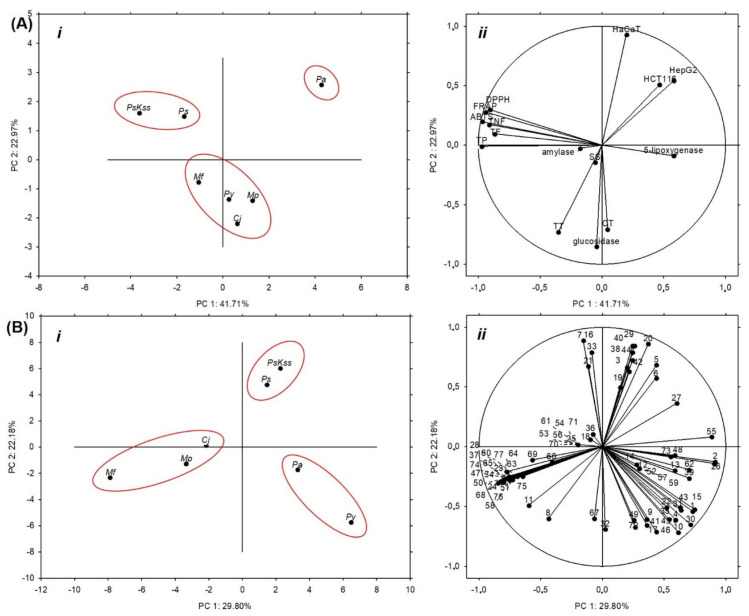
Principal component analysis of (**A**) the groups of metabolites, antioxidant capacity, cytotoxicity, antidiabetic and anti-inflammatory potential of *Rosaceae* inflorescences: (**i**) score plot separating the inflorescence samples based on the measured groups of metabolites, antioxidant capacity, cytotoxicity, and antidiabetic and anti-inflammatory potential, and (**ii**) loading plot of the measured variables; (**B**) the individual identified phenolic compounds in *Rosaceae* inflorescences: (**i**) score plot separating the inflorescence samples based on the individual identified phenolic compounds they contain, and (**ii**) the loading plot of the individual phenolics as variables. *Pa* = *P. avium*, *Ps* = *P. serrulata*, *PsKss* = *P. serrulata* ‘Kiku Shidare Zakura’, *Py* = *P. yedoensis*, *Mp* = *M. purpurea*, *Mf* = *M. floribunda*, *Cj* = *Chaenomeles japonica*, TP = total phenolics, TF = total flavonoids, TNF = total nonflavonoids, TT = total tannins, CT = condensed tannins, SS = soluble sugars, ABTS = 2,2′-azino-bis(3-ethylbenzothiazoline-6-sulfonic acid) diammonium salt, FRAP = ferric reducing antioxidant power, DPPH = 2,2-diphenyl-1-picrylhydrazyl, 1–77 = numbers related to the individual identified phenolics, as depicted in the Table 2.

The potential of *Pa* fruits to inhibit α-glucosidase is known [22]; however, there are no data on their or other *Rosaceae* inflorescences’ antidiabetic activity. In our study, *Mf* and *Cj* showed a significantly higher rate of α-glucosidase inhibition than the other samples (Figure 1B). Moreover, the inhibition percentages (86.29 ± 3.78% and 83.91 ± 0.48%, respectively) were very close to the value of the standard acarbose at the same concentration, 92.70 ± 1.21% (Figure 1). Compared to the inhibition percentages of common vegetables [32], *Rosaceae* inflorescences can justifiably be considered relevant natural α-glucosidase inhibitors. Acarbose, a pseudotetrasaccharide, is otherwise a highly effective inhibitor of intestinal α-glucosidases; however, it is not effectively absorbed into the bloodstream, but rather retained in the intestine, and may cause gastrointestinal side effects [29]. Therefore, the plant-based α-glucosidase inhibitors with lower side effects are very welcome. This indicates the high potential of *Mf* and *Cj* inflorescences to attenuate hyperglycemia, and should definitely be investigated further in *in vivo* models. For example, just recently, Kumar et al. [33] revealed that the extract of *P. amygdalus* seed coat applied to diabetic rats significantly reduced the level of blood glucose, and down-regulated hyperglycemic stress, oxidative stress and hyperlipidaemia. Moreover, they found out that the *in vivo* antidiabetic activity of the extract was accomplished via the inhibition of dipeptidyl peptidase IV (DPP-IV) protein. This is a hydrolase distributed in various tissues and the circulation, which quickly metabolizes glucagon-like peptide-1 (GLP-1). GLP-1, otherwise, maintains the blood glucose level, supporting insulin secretion and β-cell masses, reducing glucagon secretion, and changing the rate of gastric emptying [34,35]. By inhibiting the DPP-IV, the level of GLP-1 can be maintained; in this way the blood glucose level can be maintained as well. We hypothesize that *Mf* and *Cj* inflorescences’ extracts might also affect DPP-IV activity *in vivo*, and this would be good to test in future. Considering the content of phytochemicals, *Mf* had the highest concentration of total tannins among the samples, 107.85 ± 1.09 mg CE/g DW, and *Cj*—as mentioned earlier—had the highest concentration of total condensed tannins and individual identified procyanidins (Table 1, Table 2). Therefore, these groups of compounds were probably responsible for the strong α-glucosidase inhibition. Furthermore, PCA revealed that the total and condensed tannins mostly contributed to the inhibition of α-glucosidase (Figure 2Aii).

The significance of condensed tannins in α-glucosidase inhibition has also been detected in the analysis of *P. persica* pulp [13]. The individual identified compounds predominating in *Cj* we have already emphasized, and in *Mf* the predominant compounds were chlorogenic acid (8.41 ± 0.69 mg/g DW), di-caffeoylquinic acid 2, eriodictyol hexoside 1 and 2, quercetin-3-rhamnoside, quercetin-arabinofuranoside, kaempferol rhamnoside, isorhamnetin hexoside, syringetin hexoside 1, phloridzin, trilobatin, procyanidin dimers, procyanidin trimers, and procyanidin tetramer (Table 2). At the same time, *Mf* had the highest concentration of total identified compounds, total identified procyanidins, chalcones, flavonols and flavanones (Table 1). As with α-amylase, we would also like to draw attention to the identified procyanidins, especially procyanidin trimer 3, which was present in *Mf* with more than 10% of the total identified compounds in that species, and probably significantly affected the activity of α-glucosidase. In both of these species, in addition to the identified procyanidins, the predominant compound among the individual compounds was chlorogenic acid, at 8.41 ± 0.69 mg/g DW in *Mf* and 7.04 ± 0.23 mg/g DW in *Cj* (Table 2); as such, we assume that these compounds may be responsible for potent α-glucosidase inhibition. Another thing that we detected is that *Mf* and *Cj* had high concentrations of procyanidins similar to hawthorn (*Crataegus* spp.) fruits, which are—due to the preventive activity of these compounds toward oxidative stress after ischemia repercussion injury and myocardial infarction—included in the European pharmacopeia as a complementary treatment for chronic heart failure [36]. Therefore, we think, in the future, that it would be wise to test the potential of *Mf* and *Cj* inflorescences’ extracts for the prevention or alleviation of cardiovascular diseases.

### 2.4. Anti-Inflammatory Activity of Inflorescences

5-Lipoxygenase catalyses the first step in the biosynthesis of leukotrienes, which are pivotal lipid mediators of inflammation and allergy. The inhibition of this enzyme is one of the strategies to reduce inflammation, and the search for natural inhibitors of 5-lipoxygenase is very active. It is known that a fraction of *Py* bark suppressed inflammatory chemokines in human HaCaT keratinocytes, and that it might have anti-atopic dermatitis activity [11]. However, the possibility of inflammation suppression with *Rosaceae* inflorescences has not been tested so far. Therefore, this is the first report on 5-lipoxygenase inhibition by *Rosaceae* inflorescences.

In our work, *Cj* was the most efficient species in the inhibition of 5-lipoxygenase, but it was less efficient than the standard nordihydroguaiaretic acid (Figure 1C). Very recently, Turkiewicz et al. [20] tested the inhibition potential of *Chaenomeles* species and cultivars’ fruits toward 15-lipoxygenase; compared to their results, *Cj* inflorescences are much more efficient against 5-lipoxygenase, and thus present a promising biomaterial for further analysis. Our analyses showed that only *Cj* had naringenin-hexoside, isorhamnetin acetyl hexoside 1 and 2, myricetin rutinoside, syringetin acetyl hexoside 2, and procyanidin dimer 5 and 6 (Table 2), and that it had the highest content of total condensed tannins (Table 1), 5-*p*-coumaroyl-quinic acid 2, and procyanidin dimer 1, 2 and 4 (Table 2). Thus, we assume that these compounds, or at least some of them, were crucial for the anti-inflammatory activity of this sample.

### 2.5. Cytotoxic Activity of the Inflorescences

In this study, we evaluated the *in vitro* anti-proliferative effect of *Rosaceae* inflorescences on human hepatocellular (HepG2) and colorectal (HCT 116) carcinoma cells, and non-tumorigenic skin keratinocytes (HaCaT). There is evidently a variability in the cell’s metabolic response to the extracts (Table 4).

The most potent cytotoxic activity shown by *Pa* toward HCT 116 cell line IC_50_ was 261.97 ± 13.12 μg/mL, toward HepG2 IC_50_ was 300.89 ± 0.21 μg/mL, and toward HaCaT cells IC_50_ was 323.84 ± 46.61 μg/mL (Table 4). Jesus et al. [8] speculated that the high concentration of chlorogenic acid in the leaves of *Pa* might be the reason of their anticancer activity. However, among the tested samples in our work, *Pa* was not the one with the highest concentration of this acid (Table 2), and still showed the most potent anticancer activity (Table 4). Therefore, we presume that chlorogenic acid, in itself, may not be crucial for the antiproliferative activity of *Pa* inflorescences, but in combination with other compounds could act synergistically and enhance the cytotoxicity. Interestingly, *Pa* stem extracts up to the concentration of 400 μg/mL did not show cytotoxicity toward different cancer cell types, including HepG2, while extracts of the fruits revealed selectivity against colon carcinoma HCT-15 [19]. The cytotoxic effects of isolated catechins are known from both *in vitro* and *in vivo* investigations; however, in combinations with the other compounds in the extract, their effects might be different [37]. What surprised us was that *Pa* had the lowest concentrations of catechins and their oligo- and polymers (Supplementary Figure S3). The cytotoxic activity of these phenolics is well known; however, in the extracts of *Pa*, *Ps*, *PsKss* and *Py,* some chemical interactions occur between compounds which lead to unexpected effects—the highest cytotoxicity was found in samples with the lowest concentration of catechins, and this was especially emphasized with HaCaT cells, suggesting the cell-based specificity of the extracts as well. Moreover, we detected a strong negative linear correlation between the viability of HaCaT cells, and the content of total tannins (*R*^2^ = 0.780) and condensed tannins (*R*^2^ = 0.977), and the total identified flavan-3-ols (*R*^2^ = 0.952) and procyanidins (*R*^2^ = 0.990) among the *Prunus* samples (Supplementary Figure S3). We hypothesize that these compounds in some way interfere with the cytotoxic effect of *Prunus* inflorescences’ extracts toward HaCaT. When we looked at the individual components, we detected that epicatechin, procyanidin dimer 2 and procyanidin trimer 1 had strong negative correlations (*r* = −0.622, *r* = −0.810, and *r* = −0.686, respectively) with the HaCaT viability (Supplementary Table S2), so we assume that these compounds might attenuate the cytotoxicity of the *Prunus* extracts. One possible explanation could be that flavan-3-ols (catechins), and their oligomers and polymers, bind the cytotoxic components of the extracts and act as their antagonists. The higher the concentration of the total and condensed tannins, and the total identified procyanidins and flavanols in *Prunus* inflorescences, the higher the number of viable HaCaT cells. This is useful information for the possible application of the tested *Prunus* extracts in dermal wound healing.

*Ps* and *PsKss* showed similar cytotoxic potentials to *Pa* toward HaCaT cells. Cell-specific cytotoxicity levels were ascertained for *Ps* (HaCaT was most susceptible), *Mp* (HepG2 was most sensitive), and *Mf* (HCT 116 was most sensitive) (Table 4).

So far, the anticancer properties of the fruits and stems of *Pa* have been investigated against five human cancer cell lines, and only the extract of the fruits showed activity against colon carcinoma HCT-15 [19]. Because fruits contain anthocyanins, and stems do not, the presumption is that they might be the key to the anticancer properties of *Pa* fruits. The inflorescences have not been tested so far. 

A very strong positive correlation was found between cytotoxic activity against HepG2 and di-caffeoylquinic acid 3, isorhamnetin dihexoside, laricitrin glucuronide and syringetin hexoside 2 (Supplementary Table S1), all of which are predominantly present in *Pa*. As such, these compounds might be responsible for the inhibition of cell proliferation, and their cytotoxic potential should be investigated further. The cytotoxic activity toward HCT116 was very strongly correlated with di-caffeoylquinic acid 3,5-feruloylquinic acid, isorhamnetin dihexoside, laricitrin glucuronide and syringetin hexoside 2, which were again predominant in *Pa*. On the other hand, caffeic acid dihexoside was the only compound that was very strongly correlated with an antiproliferative effect on HaCaT cells (Supplementary Table S2).

### 2.6. Statistical Analysis

The principal component analysis (PCA) based on the measured groups of metabolites and the antioxidant capacity (Table 1), cytotoxicity (Table 4), and hypoglycemic and anti-inflammatory potential (Figure 1) explained 64.68% of the total variation among the samples, where PC1 accounted for 41.71% of the variance and PC2 accounted for 22.97% (Figure 2Ai). The samples were separated into three groups: *Ps* and *PsKss* formed one group; *Mp*, *Cj*, *Py* and *Mf* formed the other; and *Pa* was separated alone as the most specific sample (Figure 2Ai). The variables that mostly contributed to the group of *Ps* and *PsKss* were antioxidant capacity (ABTS, FRAP and DPPH), total phenolics, flavonoids, and nonflavonoid compounds (Figure 2Aii). The separation of *Pa* was due to the cytotoxic activity toward all of the three tested cell types. The group of *Mp*, *Cj*, *Py* and *Mf* mostly contributed tannins, soluble sugars, and the inhibition of α-glucosidase.

Based on the individual identified phenolics, the samples were similarly grouped: the only difference was in the grouping of *Py*, which was closer to *Pa*, and they grouped together (Figure 2Bi). This suggests that the representation of the individual identified phenolics and the bioactivity of the tested *Rosaceae* inflorescences are analogously distributed between all of the samples, except for *Py*. We also noticed that *Cj*, based on the measured parameters, was closer to the *Malus* than to the *Prunus* samples.

HC analysis, an algorithm that creates a dendrogram showing the hierarchical relationships between different datasets, showed the degree of similarities/dissimilarities between the samples. Based on their groups of metabolites, antioxidant capacity, cytotoxicity, and hypoglycemic and anti-inflammatory potential, *Ps* and *PsKss* were the most similar samples to each other. Other samples were close to them, while *Pa* was the most distant from all of the samples (Supplementary Figure S4A). These results indicate that greater genetic similarity does not imply a greater similarity in biological effects (Supplementary Figure S4A).

Based on their individual identified phenolic compounds, the samples were, as expected, more similar (closer) to each other (Supplementary Figure S4B); the most similar were *Pa* and *Ps*, while the most distant from them was *Py*. 

Pearson’s correlation coefficient (r) between the groups of metabolites, antioxidant capacity, cytotoxicity, and hypoglycemic and anti-inflammatory potential of the samples revealed a strong positive correlation between antioxidant capacity (ABTS, FRAP and DPPH) and the total phenolics, flavonoids and nonflavonoids (Table 3). The inhibition of α-glucosidase was strongly positively correlated with cytotoxicity toward HaCaT cells (Table 3). Cytotoxicity toward HepG2 was positively correlated with cytotoxicity toward both HCT 116 and HaCaT cells. Cytotoxic activities toward HCT 116 and HaCaT were also strongly correlated. The total tannins exhibited a strong negative correlation with cytotoxicity toward HCT 116 and HaCaT cells. This suggests that a removal of tannins from the extracts might increase the cytotoxic effect/s of the samples toward HCT 116 and HaCaT, along with their inhibition of α-glucosidase, which is worthy of further investigations. The inhibition of α-glucosidase was strongly positively correlated with the total and condensed tannins, which indicates that these compounds might be responsible for the inhibition of this enzyme. These results are in accordance with previous observations regarding the tannin effect on glucosidase activity [38].

As far as individual compounds are concerned, according to Pearson’s correlation coefficient, the only compound that was very strongly correlated with the inhibition of α-glucosidase was chlorogenic acid (Supplementary Table S2); as such, we suggest the further investigation of the antidiabetic potential of this phenolic compound. The results from all three antioxidant methods strongly correlated with caffeic acid hexoside 1, 3-caffeoylquinic acid, 4-caffeoylquinic acid, 3-*p*-coumaroylquinic acid, *p*-coumaric acid hexoside 2, catechin, quercetin-hexoside pentoside, quercetin-3-glucoside, kaempferol dihexoside and kaempferol trihexoside. Therefore, we assume that these compounds mostly contributed to the antioxidant activity of the inflorescences. The cytotoxic activity toward HepG2, HCT 116 and HaCaT strongly correlated with di-caffeoylquinic acid 3,3-feruloylquinic acid, 5-feruloylquinic acid, isorhamnetin dihexoside, laricitrin glucuronide and syringetin hexoside 2. The individual compounds that strongly correlated with the inhibition of both antidiabetic enzymes, α-amylase and α-glucosidase, were procyanidin dimer 1 and 4. With the inhibition of α-amylase, the only strong correlations were naringenin hexoside, isorhamnetin acetyl hexoside 1 and 2, myricetin rutinoside, syringetin acetyl hexoside 2, and procyanidin dimer 1, 4, 5 and 6. The inhibition of α-glucosidase strongly correlated with chlorogenic acid; procyanidin dimer 1, 2, 4; and procyanidin tetramer. 

Based on the results, we propose the consideration of *Prunus*, *Malus* and *Chaenomeles* inflorescences as low-sugar plant foods rich in polyphenolic bioactive compounds, or at least as functional additives to regular food that could improve human health. In particular, we propose *Cj* inflorescences for further *in vivo* studies of their antidiabetic and anti-inflammatory activity, and for possible use as a functional food. *Prunus* inflorescences would be excellent candidates for further analyses of the influence of monomers, oligomers and polymers of flavanols on HaCaT cells’ proliferation. Finally, *PsKss* is a material with a respectable amount of total phenolics and flavonoids that shows strong antioxidant activity and α-amylase inhibition; therefore, it is worthy of further *in vitro* and *in vivo* investigations.

## 3. Materials and Methods

### 3.1. Chemicals and Materials

All of the chemicals and reagents were supplied by Sigma Aldrich GmbH (Taufkirchen, Germany). A Gemini 3 µm C18 110A New Column 150 × 4.6 mm and a Gemini C18 4 × 3.0 mm guard column were purchased from Phenomenex (Torrance, CA, USA). The HaCaT, HCT 116 and HepG2 were obtained from the American Type Culture Collection (ATCC, Manassas, VA, USA).

The inflorescence samples of each of the variety—*Pa*, *Ps*, *PsKss*, *Mp*, *Mf* and *Cj*—were collected separately three times during April 2018 in the Botanical Garden of the Faculty of Science, University of Zagreb, Croatia. The plant material was freeze dried using an Alpha 1–2 Christ freeze-dryer, pulverised using a pestle and mortar, and then used to prepare the extracts.

### 3.2. Extraction of the Phenolic Compounds

The phenolic compounds were extracted in an ice-cold ultrasonic bath for 60 min with 70% EtOH using an equivalent of 30 mg/mL dry weight. The homogenates were centrifuged for 8 min at 9500 rpm. The supernatant was filtered through a Chromafil AO-20/25 polyamide ester filter produced by Macherey-Nagel (Düren, Germany).

### 3.3. Spectrophotometric Determination of the Phytochemicals and Antioxidant Capacity

The total phenols, flavonoids, non-flavonoids, tannins, condensed tannins and soluble sugars were determined as in Poljuha et al. [39] and Šola et al. [40,41]. The antioxidant activity of the non-hydrolyzed extracts was measured using three assays (ABTS, DPPH and FRAP), as described in Poljuha et al. [39]. Three repetitions in three independent experiments were performed for the same sample, and all of the absorbance measurements were performed with a Fluostar Optima microplate reader (BMG Labtech GmbH, Offenburg, Germany) at different wavelengths (734 nm for ABTS, 517 nm for DPPH, and 593 nm for FRAP).

### 3.4. LC-DAD-MS Analysis

The phenolic compounds were analysed on a Dionex HPLC system with a diode array detector (Thermo Scientific, San Jose, CA, USA) using Chromeleon workstation software. A Column Gemini heated at 25 °C was used. The extracts were eluted with aqueous 0.1% formic acid and 3% acetonitrile (A), and 0.1% formic acid and 3% water in absolute acetonitrile (B). All of the phenolic compounds were identified using a mass spectrometer (LTQ XL Linear Ion Trap Mass Spectrometer, Thermo Fisher Scientific, USA) with an electrospray interface (ESI) operating in negative ion mode, under the same conditions as those reported by Mikulic-Petkovsek et al. [42]. The contents of the phenolic compounds were calculated from the peak areas of the sample and the corresponding standards, and were expressed in mg/g dry weight. All of the analyses were performed in triplicate.

### 3.5. Effect of the Extracts on Antidiabetic (α-Amylase and α-Glucosidase) and Anti-Inflammatory (5-Lipoxygenase) Activity

The α-amylase inhibitory activity was tested as reported in Šola et al. [41], and the absorbance was measured at 544 nm using a microplate reader. The percentage of α-amylase inhibition at a sample concentration of 0.8 mg/mL was calculated using the following equation:

% inhibition = [100 − (A_t_ − A_tb_)/(A*c* − A*cb*)] × 100,

where A_t_ was the absorbance of the test (with amylase), A_tb_ was the absorbance of the test blank (without amylase), A*_c_* was the absorbance of the control (with amylase), and A*_cb_* was the absorbance of the control blank (without amylase). Maltose was used as a positive control.

The inhibition of α-glucosidase was measured using the pre-incubation method, as described by Salahuddin et al. [43], with slight modifications. In brief, 20 μL extract was mixed with 100 μL *p*-nitrophenyl-α-D-glucopyranoside (1 mM in 100 Mm phosphate buffer) and pre-incubated for 10 min at 37 °C. A volume of 100 μL α-glucosidase (56.6 mU/mL in phosphate buffer) was added and re-incubated for 20 min at 37 °C. The enzyme reaction activity was terminated by the addition of 500 μL Na_2_CO_3_ (1 M). The absorbance was measured at 405 nm using a microplate reader. The enzyme inhibitory activity at a sample concentration of 0.55 mg/mL was calculated similarly to that for the α-amylase inhibition. Acarbose was used as a positive control. 

The inhibition of the 5-lipoxygenase activity was assessed according to El Euch et al. [4]. In brief, in 350 µL phosphate buffer (pH 7.4) containing Na_2_HPO_4_, KH_2_PO_4_ and NaCl, 40 µL lipoxygenase enzyme (500 U) from Glycine max was mixed with 120 µL 3.5 mM linoleic acid and 40 µL 20 mg/mL inflorescence extracts. The mixture was homogenized using a vortex mixer and incubated for 10 min at 25 °C. The absorbance was measured in triplicate at 234 nm using a NanodropTM 2000c (Thermo Scientific). The enzyme inhibitory activity at a sample concentration of 1.45 mg/mL was calculated from the following equation:

% inhibition = [(A_con_ − A_background_) − (A_sample_ − A_background_) × 100%/(A_con_ − A_background_)]

where A_con_ = the absorbance of the control, A_background_ = the absorbance of the background (blank), and A_sample_ = the absorbance of the sample. Nordihydroguaiaretic acid was used as a positive control.

### 3.6. In Vitro Antiproliferative Activity

The *in vitro* antiproliferative activity (IC_50_ expressed in μg/mL) was determined for HepG2 (HB-8065), HCT 116 (CCL-247) and HaCaT (CVCL-0038) cells using an MTT assay, which is a quantitative colorimetric assay for the measurement of cell survival and proliferation, as described in Šola et al. [41]. In brief, 4 × 10^3^ cells were seeded in 96-well plates, and then treated 24 h later with different concentrations of the extracts diluted in growth medium. As a control, the same dilution of 70% ethanol in growth medium was prepared and incubated with the cells. After 72 h, the treatment was removed, 1 × MTT was added, and the cells were incubated for 4 h in the growth conditions, followed by the addition of dimethyl sulfoxide and 20 min incubation with gentle mixing. The absorbance was measured at λ = 570 nm with an ELISA microplate reader (LabSystem Multiskan MS, Artisan Technology group, Champaign, IL, USA).

### 3.7. Statistical Analysis

The data obtained were statistically processed in the Statistica 13.1 program (Stat Soft Inc., Palo Alto, CA, USA). All of the experiments were performed in triplicate. The comparison of the samples’ means was carried out using one-way variance analysis (ANOVA) and Duncan’s New Multiple Range Test (DNMRT). The statistically significant values were those that differed at the *p* ≤ 0.05 level. Principal component analysis (PCA) and hierarchical clustering (HC) were performed to evaluate how close the samples were according to the given parameters. The Pearson’s correlation coefficients between the phytochemical content and bioactivities of the inflorescences were calculated.

## 4. Conclusions

This is the first study that characterised the phytochemical profile, *in vitro* antioxidant activity, and antidiabetic, anti-inflammatory and antiproliferative potential of inflorescences of *Pa*, *Ps*, *PsKss*, *Mp*, *Mf* and *Cj*. Moreover, for the first time, *Rosaceae* inflorescences were screened for their antidiabetic and anti-inflammatory activity. The results showed that these inflorescences are a valuable source of phenolics with significant biological activities. The highest amount of total phenolics, flavonoids and nonflavonoids, and the highest antioxidant capacity measured by all three methods was recorded in *PsKss*. Besides *Mp*, this species also contained the lowest concentration of soluble sugars. In addition, the sugar content in the inflorescences was lower than those in the more commonly used fruits. Among the tested *Malus* species, *Mf* inflorescences had significantly higher total phenolics, flavonoids and tannins, as well as identified polyphenolic compounds, and showed higher antioxidant activity and α-glucosidase inhibition than *Mp*. The sample that showed the most effective inhibition of both antidiabetic enzymes and anti-inflammatory 5-lipoxygenase was *Cj*. Therefore, we suggest further *in vitro* and *in vivo* analyses of the antidiabetic and anti-inflammatory potential of *Cj* inflorescences. Beside *Cj*, *PsKss* showed strong α-amylase inhibition, and the strong inhibition of α-glucosidase *Mf*. The cytotoxic activity was both species- and cell-type-specific. The most effective in the inhibition of the growth and proliferation of HepG2, HCT 116 and HaCaT cells was *Pa*. The antiproliferative effect of extracts from *Prunus* inflorescences was negatively correlated with the concentration of the total and condensed tannins, identified procyanidins (especially procyanidin dimer 2 and procyanidin trimer 1) and flavanols (especially epicatechin). The inhibition of α-glucosidase showed a strong negative correlation with the total tannins. The results in this work encourage further *in vivo* studies of these matrices, and suggest innovations for future-oriented diets and food production, such as the incorporation of *Prunus*, *Malus* and *Chaenomeles* inflorescences’ extracts as dietary supplements and functional ingredients.

## Figures and Tables

**Table 1:** Concentration (in mg/g dry weight) of the total phenolics (TP), total flavonoids (TF), total nonflavonoids (TNF), total tannins (TT), condensed tannins (CT) and soluble sugars (SS), and the antioxidant capacity (ABTS, FRAP and DPPH) of *Rosaceae* inflorescences.

	*Prunus avium*	*Prunus serrulata*	*Prunus serrulata* ‘Kiku Shidare Zakura’	*Prunus yedoensis*	*Malus purpurea*	*Malus floribunda*	*Chaenomeles japonica*
TP (mg GAE/g DW)	27.83 ± 0.69 f	46.84 ± 0.75 b	53.12 ± 0.79 a	37.31 ± 0.48 e	40.29 ± 0.64 c	46.74 ± 0.93 b	39.42 ± 0.97 d
TF (mg CE/g DW)	13.57 ± 0.84 f	32.35 ± 2.13 b	38.89 ± 4.04 a	25.78 ± 1.03 d	13.43 ± 0.82 f	23.83 ± 0.88 e	29.45 ± 0.65 c
TNF (mg GAE/g DW)	16.40 ± 0.91 g	29.35 ± 0.83 b	31.32 ± 0.71 a	22.21 ± 0.86 e	23.52 ± 0.56 d	28.85 ± 0.58 c	18.73 ± 0.48 f
TT (mg CE/g DW)	27.26 ± 0.22 g	59.44 ± 1.32 f	71.59 ± 0.33 d	83.55 ± 0.55 b	64.32 ± 0.45 e	107.85 ± 1.09 a	80.27 ± 0.33 c
CT (mg CE/g DW)	4.25 ± 0.33 e	7.74 ± 1.02 d	6.99 ± 0.17 d	16.52 ± 0.01 b	15.45 ± 0.10 b	10.98 ± 0.24 c	51.68 ± 0.38 a
SS (mg SE/g DW)	3.37 ± 0.06 c	3.04 ± 0.05 d	2.41 ± 0.10 e	8.61 ± 0.12 a	1.56 ± 0.08 f	3.58 ± 0.06 b	3.27 ± 0.07 c
ABTS (mg TE/g DW)	22.86 ± 4.61 e	49.41 ± 7.23 b	61.32 ± 5.84 a	36.63 ± 4.32 c	28.78 ± 2.52 d	47.78 ± 6.26 b	35.05 ± 4.25 c
FRAP (mg TE/g DW)	27.89 ± 0.60 g	51.68 ± 0.12 b	58.06 ± 0.78 a	40.28 ± 1.18 d	29.12 ± 0.83 f	44.36 ± 0.85 c	36.36 ± 1.50 e
DPPH (mg TE/g DW)	25.47 ± 2.57 d	52.95 ± 4.22 b	69.42 ± 3.27 a	39.21 ± 4.86 c	25.10 ± 3.25 d	40.38 ± 3.4 c	39.61 ± 3.95 c

Values represent the mean ± standard deviation of three replicates. Different letters indicate a significant difference among the values in a row (ANOVA, Duncan test, *p* ≤ 0.05). GAE = gallic acid equivalent, CE = catechin equivalent, SE = sucrose equivalent, TE = trolox equivalent.

**Table 2:** Concentration (mg/g DW ± SD) of the individual phenolic compounds in *Rosaceae* inflorescences.

		*Prunus avium*	*Prunus serrulata*	*Prunus serrulata* ‘Kiku Shidare Zakura’	*Prunus yedoensis*	*Malus purpurea*	*Malus floribunda*	*Chaenomeles japonica*
1	Gallic acid	0.28 ± 0.06 b	0.15 ± 0.04 c	0.06 ± 0.01 d	0.38 ± 0.03 a	0.17 ± 0.04 c	0.03 ± 0.01 d	nd
	Total identified hydroxybenzoic acids	0.28 ± 0.06 b	0.15 ± 0.04 c	0.06 ± 0.01 d	0.38 ± 0.03 a	0.17 ± 0.04 c	0.03 ± 0.01 d	nd
2	Caffeic acid	1.95 ± 0.27 c	1.25 ± 0.14 c	2.50 ± 0.08 b	3.90 ± 0.10 a	nd	nd	0.53 ± 0.08 d
3	Caffeic acid hexoside 1	0.14 ± 0.03 b	0.39 ± 0.06 b	15.48 ± 2.23 a	0.10 ± 0.01 b	0.05 ± 0.01 b	0.23 ± 0.02 b	0.75 ± 0.04 b
4	Caffeic acid hexoside 2	nd	nd	nd	5.62 ± 0.25 a	nd	nd	0.02 ± 0.00 b
5	Caffeic acid dihexoside	0.29 ± 0.01 b	0.19 ± 0.04 c	0.41 ± 0.02 a	nd	nd	nd	nd
6	3-caffeoylquinic acid	nd	0.36 ± 0.04 b	1.70 ± 0.25 a	0.54 ± 0.03 b	nd	nd	0.02 ± 0.00 c
7	4-caffeoylquinic acid	nd	0.27 ± 0.08 a	0.35 ± 0.02 a	nd	0.13 ± 0.06 b	0.15 ± 0.02 b	0.07 ± 0.01 b
8	5-caffeoylquinic acid 1	1.59 ± 0.18 d	0.59 ± 0.17 e	0.47 ± 0.02 e	5.75 ± 0.15 c	1.41 ± 0.30 d	8.41 ± 0.69 a	7.04 ± 0.23 b
9	5-caffeoylquinic acid 2	0.43 ± 0.36 a	nd	nd	0.27 ± 0.01 a	0.31 ± 0.13 a	nd	0.14 ± 0.02 a
10	di-caffeoylquinic acid 1	3.12 ± 0.08 b	0.15 ± 0.03 c	0.31 ± 0.02 c	7.06 ± 0.82 a	0.65 ± 0.14 c	0.50 ± 0.03 c	2.83 ± 0.30 b
11	di-caffeoylquinic acid 2	0.13 ± 0.01 b	nd	nd	0.29 ± 0.01 b	0.16 ± 0.06 b	1.02 ± 0.15 a	nd
12	di-caffeoylquinic acid 3	0.17 ± 0.03 a	nd	nd	nd	nd	nd	nd
13	3-feruloylquinic acid	0.24 ± 0.04 a	0.03 ± 0.00 c	0.05 ± 0.00 bc	0.08 ± 0.00 b	nd	nd	0.004 ± 0.001 c
14	5-feruloylquinic acid	0.26 ± 0.03 a	0.06 ± 0.01 d	0.11 ± 0.00 bc	0.09 ± 0.01 cd	0.01 ± 0.00 e	0.13 ± 0.04 b	0.01 ± 0.00 e
15	3-*p*-coumaroylquinic acid	0.52 ± 0.08 b	0.14 ± 0.02 d	0.21 ± 0.01 c	0.73 ± 0.03 a	0.03 ± 0.01 e	0.15 ± 0.01 cd	0.001 ± 0.000 e
16	4-*p*-coumaroylquinic acid	nd	0.27 ± 0.08 a	0.35 ± 0.02 a	nd	0.13 ± 0.06 b	0.15 ± 0.02 b	0.07 ± 0.02 b
17	5-*p*-coumaroylquinic acid 1	0.39 ± 0.02 a	0.09 ± 0.01 d	0.07 ± 0.00 d	0.33 ± 0.03 b	0.10 ± 0.01 d	0.19 ± 0.03 c	0.12 ± 0.03 d
18	5-*p*-coumaroylquinic acid 2	0.03 ± 0.00 c	0.09 ± 0.01 b	0.08 ± 0.01 bc	0.10 ± 0.01 b	0.07 ± 0.06 bc	0.06 ± 0.02 bc	0.25 ± 0.04 a
19	*p*-coumaric acid hexoside 1	0.23 ± 0.06 b	0.21 ± 0.04 b	0.31 ± 0.01 a	0.09 ± 0.00 c	0.04 ± 0.01 cd	0.22 ± 0.04 b	0.001 ± 0.000 d
20	*p*-coumaric acid hexoside 2	0.37 ± 0.06 b	1.42 ± 0.15 a	1.34 ± 0.05 a	0.21 ± 0.01 c	0.02 ± 0.00 d	0.11 ± 0.01 cd	0.13 ± 0.00 cd
	Total identified hydroxycinnamic acids	9.87 ± 0.08 b	5.51 ± 0.06 c	23.70 ± 0.18 a	25.16 ± 0.10 a	3.12 ± 0.06 d	11.31 ± 0.09 b	11.93 ± 0.05 b
21	Catechin	0.23 ± 0.04 e	2.61 ± 0.27 a	2.46 ± 0.09 a	0.91 ± 0.02 d	nd	1.99 ± 0.16 b	1.67 ± 0.06 c
22	Epicatechin	1.10 ± 0.15 d	0.91 ± 0.10 d	1.81 ± 0.06 b	6.92 ± 0.18 a	1.18 ± 0.18 d	1.38 ± 0.22 c	0.40 ± 0.06 e
	Total identified flavanols	1.33 ± 0.09 e	3.52 ± 0.18 cd	4.27 ± 0.07 b	7.83 ± 0.10 a	1.18 ± 0.18 e	3.38 ± 0.19 bc	2.08 ± 0.06 de
23	Eriodictyol hexoside 1	0.03 ± 0.00 b	nd	nd	nd	0.63 ± 0.04 b	2.92 ± 0.86 a	nd
24	Eriodictyol hexoside 2	nd	nd	nd	nd	0.35 ± 0.09 b	1.02 ± 0.15 a	nd
25	Naringenin hexoside	nd	nd	nd	nd	nd	nd	0.62 ± 0.04 a
	Total identified flavanones	0.03 ± 0.00 b	nd	nd	nd	0.98 ± 0.06 b	3.93 ± 0.50 a	0.62 ± 0.04 b
26	Quercetin-glycoside	0.18 ± 0.01 c	0.13 ± 0.01 d	0.21 ± 0.02 b	0.36 ± 0.02 a	nd	nd	nd
27	Quercetin-3-rutinoside	2.78 ± 0.28 b	0.58 ± 0.10 d	4.79 ± 0.13 a	1.93 ± 0.03 c	0.08 ± 0.03 e	0.21 ± 0.03 e	0.72 ± 0.04 d
28	Quercetin-3-rhamnoside hexoside	nd	nd	nd	nd	0.58 ± 0.21 a	0.41 ± 0.06 a	nd
29	Quercetin-hexoside pentoside	nd	0.25 ± 0.08 b	0.59 ± 0.01 a	nd	nd	nd	nd
30	Quercetin-rhamnoside dihexoside 1	0.15 ± 0.01 b	nd	nd	0.20 ± 0.00 a	nd	nd	nd
31	Quercetin-rhamnoside dihexoside 2	nd	0.02 ± 0.01 c	0.05 ± 0.00 b	0.47 ± 0.01 a	nd	nd	nd
32	Quercetin-3-galactoside	0.26 ± 0.02 c	nd	nd	0.98 ± 0.01 b	1.21 ± 0.27 a	0.309 ± 0.070 c	0.17 ± 0.01 c
33	Quercetin-3-glucoside	0.03 ± 0.00 d	0.19 ± 0.02 a	0.12 ± 0.01 b	nd	0.02 ± 0.00 d	0.089 ± 0.004 c	0.02 ± 0.00 d
34	Quercetin-3-rhamnoside	nd	nd	nd	nd	2.26 ± 0.38 b	4.31 ± 0.51 a	nd
35	Quercetin-3-xyloside	0.01 ± 0.00 e	0.02 ± 0.00 de	0.06 ± 0.00 c	0.56 ± 0.04 a	0.09 ± 0.01 b	0.05 ± 0.01 cd	0.02 ± 0.00 de
36	Quercetin-arabinofuranoside	0.13 ± 0.04 c	0.15 ± 0.02 c	0.25 ± 0.03 b	0.17 ± 0.00 c	0.03 ± 0.01 d	0.31 ± 0.04 a	0.004 ± 0.000 d
37	Quercetin-arabinopyranoside	0.01 ± 0.00 c	nd	nd	nd	2.07 ± 0.20 a	1.61 ± 0.05 b	nd
38	Quercetin-acetyl hexoside 1	nd	4.00 ± 0.40 a	1.53 ± 0.07 b	0.22 ± 0.00 c	nd	nd	nd
39	Quercetin-acetyl hexoside 2	nd	0.14 ± 0.02 b	0.08 ± 0.00 c	0.35 ± 0.02 a	nd	nd	nd
40	Kaempferol trihexoside	nd	1.27 ± 0.11 a	0.81 ± 0.08 b	nd	nd	nd	nd
41	Kaempferol-3-rutinoside	0.92 ± 0.02 a	0.06 ± 0.00 f	0.05 ± 0.01 f	0.54 ± 0.01 b	0.37 ± 0.08 c	0.24 ± 0.03 d	0.15 ± 0.01 e
42	Kaempferol acetyl hexoside 1	nd	1.50 ± 0.15 a	0.32 ± 0.01 b	0.09 ± 0.01 a	nd	nd	nd
43	Kaempferol acetyl hexoside 2	nd	0.07 ± 0.01 a	nd	0.33 ± 0.04 a	nd	nd	nd
44	Kaempferol dihexoside	nd	0.12 ± 0.01 b	0.44 ± 0.03 a	nd	nd	nd	nd
45	Kaempferol pentoside 1	nd	nd	nd	1.34 ± 0.09 a	nd	nd	nd
46	Kaempferol pentoside 2	nd	nd	nd	0.12 ± 0.01 a	nd	nd	nd
47	Kaempferol rhamnoside	0.06 ± 0.01 b	nd	nd	nd	0.32 ± 0.08 b	4.43 ± 0.63 a	nd
48	Kaempferol hexoside 1	0.01 ± 0.00 e	0.20 ± 0.02 b	0.08 ± 0.00 d	0.27 ± 0.02 a	0.03 ± 0.01 e	0.02 ± 0.00 e	0.14 ± 0.01 c
49	Kaempferol hexoside 2	0.69 ± 0.23 a	nd	nd	0.38 ± 0.01 b	0.01 ± 0.00 c	0.34 ± 0.04 b	nd
50	Kaempferol rhamnosyl hexoside	nd	nd	nd	nd	0.02 ± 0.01 b	0.22 ± 0.03 a	nd
51	Isorhamnetin hexoside	nd	0.01 ± 0.00 c	0.04 ± 0.00 c	0.04 ± 0.00 c	0.28 ± 0.04 b	3.12 ± 0.21 a	0.22 ± 0.00 b
52	Isorhamnetin dihexoside	0.31 ± 0.02 a	nd	nd	nd	nd	nd	nd
53	Isorhamnetin acetyl hexoside 1	nd	nd	nd	nd	nd	nd	0.98 ± 0.12 a
54	Isorhamnetin acetyl hexoside 2	nd	nd	nd	nd	nd	nd	0.04 ± 0.00 a
55	Isorhamnetin-3-rutinoside	0.02 ± 0.00 a	0.01 ± 0.00 c	0.02 ± 0.00 b	0.02 ± 0.00 b	nd	nd	nd
56	Myricetin rutinoside	nd	nd	nd	nd	nd	nd	0.004 ± 0.000 a
57	Laricitrin glucuronide	0.07 ± 0.01 a	nd	nd	nd	nd	nd	nd
58	Syringetin hexoside 1	0.02 ± 0.00 b	nd	nd	nd	0.30 ± 0.03 b	5.18 ± 0.62 a	nd
59	Syringetin hexoside 2	0.29 ± 0.10 a	nd	nd	nd	nd	nd	nd
60	Syringetin acetyl hexoside 1	nd	nd	nd	nd	1.61 ± 0.41 a	0.78 ± 0.14 b	0.30 ± 0.02 b
61	Syringetin acetyl hexoside 2	nd	nd	nd	nd	nd	nd	0.07 ± 0.00 a
	Total identified flavonols	5.93 ± 0.05 b	8.71 ± 0.06 b	9.42 ± 0.03 b	8.37 ± 0.02 b	9.29 ± 0.11 b	21.60 ± 0.16 a	2.84 ± 0.02 c
62	Apigenin hexoside	nd	0.02 ± 0.00 b	0.01 ± 0.00 b	0.04 ± 0.00 a	nd	nd	nd
	Total identified flavones	nd	0.02 ± 0.00 b	0.01 ± 0.00 b	0.04 ± 0.00 a	nd	nd	nd
63	Phloretin xylosylglucoside	nd	nd	nd	nd	0.18 ± 0.03 a	0.09 ± 0.02 b	nd
64	Phloridzin	nd	nd	nd	nd	5.14 ± 0.74 a	5.23 ± 0.52 a	nd
65	Trilobatin	nd	nd	nd	nd	0.30 ± 0.08 b	1.47 ± 0.20 a	nd
	Total identified chalcones	nd	nd	nd	nd	5.61 ± 0.28 a	6.80 ± 0.24 a	nd
66	Procyanidin dimer 1	nd	0.33 ± 0.03 c	0.52 ± 0.03 c	1.24 ± 0.04 c	1.45 ± 0.42 bc	2.52 ± 0.36 b	7.90 ± 1.48 a
67	Procyanidin dimer 2	nd	nd	nd	5.07 ± 0.19 a	0.75 ± 0.31 c	2.93 ± 0.47 b	5.30 ± 0.27 a
68	Procyanidin dimer 3	nd	nd	nd	nd	0.02 ± 0.01 b	3.43 ± 0.40 a	0.36 ± 0.02 b
69	Procyanidin dimer 4	nd	nd	nd	nd	0.03 ± 0.01 c	3.43 ± 0.39 b	6.31 ± 0.25 a
70	Procyanidin dimer 5	nd	nd	nd	nd	nd	nd	1.21 ± 0.08 a
71	Procyanidin dimer 6	nd	nd	nd	nd	nd	nd	0.50 ± 0.23 a
72	Procyanidin trimer 1	nd	1.18 ± 0.09 d	0.86 ± 0.05 d	11.05 ± 0.30 a	2.15 ± 0.31 c	4.76 ± 0.39 b	0.02 ± 0.00 e
73	Procyanidin trimer 2	nd	2.63 ± 0.18 c	2.68 ± 0.16 c	5.97 ± 0.55 a	0.12 ± 0.05 d	0.07 ± 0.01 d	4.52 ± 0.16 b
74	Procyanidin trimer 3	nd	nd	nd	nd	0.42 ± 0.04 b	8.66 ± 1.08 a	0.02 ± 0.00 b
75	Procyanidin trimer 4	nd	nd	nd	nd	2.33 ± 0.58 a	1.72 ± 0.28 a	nd
76	Procyanidin trimer 5	nd	nd	nd	nd	nd	4.10 ± 1.60 a	nd
77	Procyanidin tetramer	nd	nd	nd	nd	nd	4.11 ± 1.60 a	2.89 ± 0.18 a
	Total identified condensed tannins	nd	4.14 ± 0.10 c	4.06 ± 0.08 c	23.33 ± 0.27 b	7.27 ± 0.22 c	35.73 ± 0.66 a	29.03 ± 0.27 b
	Total identified compounds	16.44 ± 0.06 d	22.05 ± 0.07 d	41.54 ± 0.06 c	65.10 ± 0.09 b	27.62 ± 0.14 d	82.77 ± 0.26 a	46.49 ± 0.09 c

Values represent the mean ± standard deviation of three replicates. Different letters indicate a significant difference among the values in a row (ANOVA, Duncan test, *p* ≤ 0.05); nd = not detected.

**Table 3:** Pearson’s correlation coefficient (r) between the groups of metabolites, antioxidant capacity, cytotoxicity, and hypoglycemic and anti-inflammatory potential of *Rosaceae* inflorescences.

	TP	TF	TNF	TT	CT	SS	ABTS	FRAP	DPPH	HepG2	HCT116	HaCaT	α-Amyl	α-Glucos	5-Lipoxy
**TP**	1.000														
**TF**	**0.761**	1.000													
**TNF**	**0.938**	**0.629**	1.000												
**TT**	0.471	0.014	0.333	1.000											
**CT**	−0.065	0.178	−0.367	0.314	1.000										
**SS**	0.194	−0.210	0.251	0.262	0.045	1.000									
**ABTS**	**0.937**	**0.875**	**0.908**	0.160	−0.140	0.115	1.000								
**FRAP**	**0.873**	**0.913**	**0.862**	0.016	−0.176	−0.022	**0.979**	1.000							
**DPPH**	**0.830**	**0.952**	**0.762**	−0.015	−0.094	−0.170	**0.945**	**0.969**	1.000						
**HepG2**	−0.459	−0.567	−0.396	−0.336	−0.353	0.059	−0.451	−0.489	−0.393	1.000					
**HCT116**	−0.416	−0.353	−0.327	**−0.602**	−0.171	0.454	−0.250	−0.274	−0.265	**0.689**	1.000				
**HaCaT**	−0.148	−0.067	−0.029	**−0.670**	−0.512	−0.059	−0.004	0.050	0.104	**0.729**	**0.623**	1.000			
**α-amyl**	0.169	0.458	−0.116	−0.038	0.585	−0.064	0.237	0.191	0.377	0.146	0.234	0.117	1.000		
**α-glucos**	0.077	−0.083	−0.101	**0.645**	**0.627**	0.429	−0.065	−0.205	−0.213	−0.312	−0.121	**−0.786**	0.263	1.000	
**5-lipoxy**	−0.345	0.020	−0.371	−0.530	0.431	0.318	−0.172	−0.114	−0.152	−0.077	0.534	0.154	0.232	0.011	1.000

Values in bold represent strong (0.60–0.79) and very strong (0.80–1.00) correlations.

**Table 4:** *In vitro* antiproliferative activity (IC_50_ expressed in μg/mL) of *Rosaceae* inflorescence exstracts tested on hepatocellular carcinoma (HepG2), colorectal cancer (HCT 116) and keratinocyte (HaCaT) cell lines.

	Cell Type (IC_50_ μg/mL)		
	HepG2	HCT 116	HaCaT
*Prunus avium*	300.89 ± 0.21 c A	261.97 ± 13.12 c A	323.84 ± 46.61 c A
*Prunus serrulata*	473.59 ± 35.69 ab A	517.42 ± 37.10 a A	377.66 ± 34.85 bc B
*Prunus serrulata* 2018Kiku Shidare Zakura’	409.71 ± 103.52 b A	464.01 ± 57.31 a A	385.20 ± 7.27 bc A
*Prunus yedoensis*	508.09 ± 26.28 a A	537.92 ± 43.0 a A	521.64 ± 67.29 a A
*Malus purpurea*	386.2 ± 19.92 b B	539.66 ± 45.19 a A	461.39 ± 71.56 ab AB
*Malus floribunda*	445.78 ± 27.42 ab A	361.83 ± 31.19 b B	459.28 ± 43.69 ab A
*Chaenomeles japonica*	452.48 ± 15.18 ab A	470.66 ± 48.16 a A	473.27 ± 92.54 ab A

Values represent the mean ± standard deviation of three replicates. Different small letters indicate a significant difference among the values in a column, and different capital letters indicate a significant difference among the values in a row (ANOVA, Duncan test, *p* ≤ 0.05).

## Data Availability

Not applicable.

## Supplementary Materials

The following are available online at https://www.mdpi.com/article/10.3390/plants11030271/s1, Figure S1: The representative chromatograms, at 280 and 350 nm, of (A) *Prunus avium*, (B) *P. serrulata*, (C) *P. serrulata* ‘Kiku Shidare Zakura’, (D) *P. yedoensis*, (E) *Malus purpurea*, (F) *M. floribunda* and (G) *Chaenomeles japonica*. The numbers above the peaks correspond to the numbers given in the Table 2. Figure S2: (A) the number of identified phenolic compounds, (B) the number of compounds with a predominant concentration, and (C) the proportion of predominantly represented compounds (%) in *Rosaceae* inflorescences. *Pa* = *P. avium*, *Ps* = *P. serrulata*, *PsKss* = *P. serrulata* ‘Kiku Shidare Zakura’, *Py* = *P. yedoensis*, *Mp* = *M. purpurea*, *Mf* = *M. floribunda*, *Cj* = *Chaenomeles japonica*. Figure S3: Linear correlation between the HaCaT cells’ viability and the amount of (A) total tannins, (B) total condensed tannins, (C) total identified procyanidins and (D) total identified flavanols in different *Prunus* varieties’ inflorescences. CE = catechin equivalent. Figure S4: Hierarchical clustering, expressed as Euclidean distance, of *Rosaceae* inflorescences (*Pa, Pss, PsKss, Py, Mp, Mf, Cj*) based on their (A) groups of metabolites, antioxidant capacity, cytotoxicity, antidiabetic and anti-inflamatory potential; and (B) identified individual phytochemicals. *Pa* = *P. avium*, *Ps* = *P. serrulata*, *PsKss* = *P. serrulata* ‘Kiku Shidare Zakura’, *Py* = *P. yedoensis*, *Mp* = *M. purpurea*, *Mf* = *M. floribunda*, *Cj* = *Chaenomeles japonica*. a, a1-a1-1-1-1-1 = clusters. Table S1: Identification of phenolic compounds in *Rosaceae* inflorescences in negative ionization with LC-DAD-MS and MS^2^/MS^3^. Table S2: Pearson’s correlation coefficient (r) between the individual identified compounds, antioxidant capacity, cytotoxicity, and hypoglycemic and anti-inflammatory potential of *Rosaceae* inflorescences.
