# Supplementary material for: Biopotential of Underutilized Rosaceae Inflorescences: LC-DAD-MS Phytochemical Profiles Associated with Antioxidant, Antidiabetic, Anti-Inflammatory and Antiproliferative Activity In Vitro
Source: Plants (Basel). 2022 Jan 20;11(3):271. doi: 10.3390/plants11030271 (PMC8838311; doi:10.3390/plants11030271)
Supplement: Supplementary file 1 [file plants-11-00271-s001.zip › Table S2.pdf]

Supplementary Table S2. Identification of phenolic compounds in *Rosaceae* inflorescences in negative ionization with LC-DAD-MS and MS^2/MS^3.

		$[M-H]^-$ *(m/z)*	MS^2 *(m/z)*	MS^3 *(m/z)*
1	Gallic acid	169	125	
2	Caffeic acid	179	135	
3	Caffeic acid hexoside 1	341	179	135
4	Caffeic acid hexoside 2	341	179	135
5	Caffeic acid dihexoside	341	179	135
6	3-caffeoylquinic acid	353	191, 179, 135	173, 127, 85
7	4-caffeoylquinic acid	353	173, 179	
8	5-caffeoylquinic acid 1	353	191, 179	127, 93, 85
9	5-caffeoylquinic acid 2	353	191,179,135	
10	di-caffeoylquinic acid 1	515	353	191, 179
11	di-caffeoylquinic acid 2	515	353	191, 179
12	di-caffeoylquinic acid 3	515	353	191, 179
13	3-feruloylquinic acid	367	193,134	149
14	5-feruloylquinic acid	367	191	173, 127, 85
15	3-*p*-coumaroylquinic acid	337	163	
16	4-*p*-coumaroylquinic acid	337	173, 163, 155, 137, 191	
17	5-*p*-coumaroylquinic acid 1	337	191, 173, 163, 155	
18	5-*p*-coumaroylquinic acid 2	337	191, 173, 163	
19	*p*-coumaric acid hexoside 1	325	163	
20	*p*-coumaric acid hexoside 2	325	163	
21	Catechin	289	245	
22	Epicatechin	289	245	

23	Eriodictyol hexoside 1	449	287	
24	Eriodictyol hexoside 2	449	287	
25	Naringenin hexoside	433	271	
26	Quercetin-glycoside	639	463	301
27	Quercetin-3-rutinoside	609	301	
28	Quercetin-rhamnoside hexoside	609	301	
29	Quercetin-hexoside pentoside	595	433	301
30	Quercetin-rhamnoside dihexoside 1	771	625	463, 301
31	Quercetin-rhamnoside dihexoside 2	771	625	463, 301
32	Quercetin-3-galactoside	463	301	
33	Quercetin-3-glucoside	463	301	
34	Quercetin-3-rhamnoside	447	301	
35	Quercetin-3-xyloside	433	301	
36	Quercetin-arabinofuranoside	433	301	
37	Quercetin-arabinopyranoside	433	301	
38	Quercetin-acetyl hexoside 1	505	463, 301	
39	Quercetin-acetyl hexoside 2	505	463, 301	
40	Kaempferol trihexoside	771	609	447, 285
41	Kaempferol-3-rutinoside	593	285	
42	Kaempferol acetyl hexoside 1	489	285	
43	Kaempferol acetyl hexoside 2	489	285	
44	Kaempferol dihexoside	609	447	285
45	Kaempferol pentoside 1	417	285	
46	Kaempferol pentoside 2	417	285	
47	Kaempferol rhamnoside	431	285	
48	Kaempferol hexoside 1	447	285	
49	Kaempferol hexoside 2	447	285	

50	Kaempferol rhamnosyl hexoside	593	447	285
51	Isorhamnetin hexoside	477	315	
52	Isorhamnetin dihexoside	639	477	315
53	Isorhamnetin acetyl hexoside 1	519	477	315
54	Isorhamnetin acetyl hexoside 2	519	477	315
55	Isorhamnetin-3-rutinoside	623	315	
56	Myricetin-3-rutinoside	625	317	
57	Laricitrin glucuronide	507	331	
58	Syringetin hexoside 1	507	345	
59	Syringetin hexoside 2	507	345	
60	Syringetin acetyl hexoside 1	549	345	
61	Syringetin acetyl hexoside 2	549	345	
62	Apigenin hexoside	431	269	
63	Phloretin xylosylglucoside	567	273	
64	Phloridzin	435	273	
65	Trilobatin	435	273	
66	Procyanidin dimer 1	577	451, 425, 407, 289	
67	Procyanidin dimer 2	577	451, 425, 407, 289	
68	Procyanidin dimer 3	577	451, 425, 407, 289	
69	Procyanidin dimer 4	577	451, 425, 407, 289	
70	Procyanidin dimer 5	577	451, 425 407, 289	
71	Procyanidin dimer 6	577	451, 425, 407, 289	
72	Procyanidin trimer 1	865	577, 451, 425, 407, 289	
73	Procyanidin trimer 2	865	577, 451, 425, 407, 289	
74	Procyanidin trimer 3	865	577, 451, 425, 407, 289	
75	Procyanidin trimer 4	865	577, 451, 425, 407, 289	
76	Procyanidin trimer 5	865	577, 451, 425, 407, 289	

77	Procyanidin tetramer	1153	1135, 1027, 983, 865, 863, 577, 575	
