# Supplementary figures and images for: Biopotential of Underutilized Rosaceae Inflorescences: LC-DAD-MS Phytochemical Profiles Associated with Antioxidant, Antidiabetic, Anti-Inflammatory and Antiproliferative Activity In Vitro
Source: Plants (Basel). 2022 Jan 20;11(3):271. doi: 10.3390/plants11030271 (PMC8838311; doi:10.3390/plants11030271)

(A)

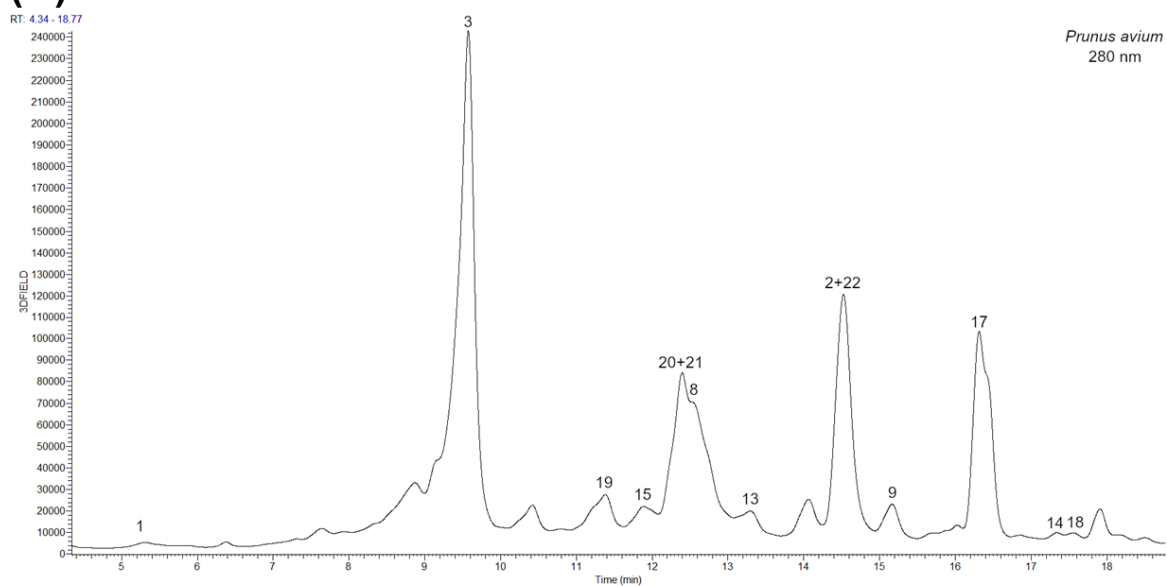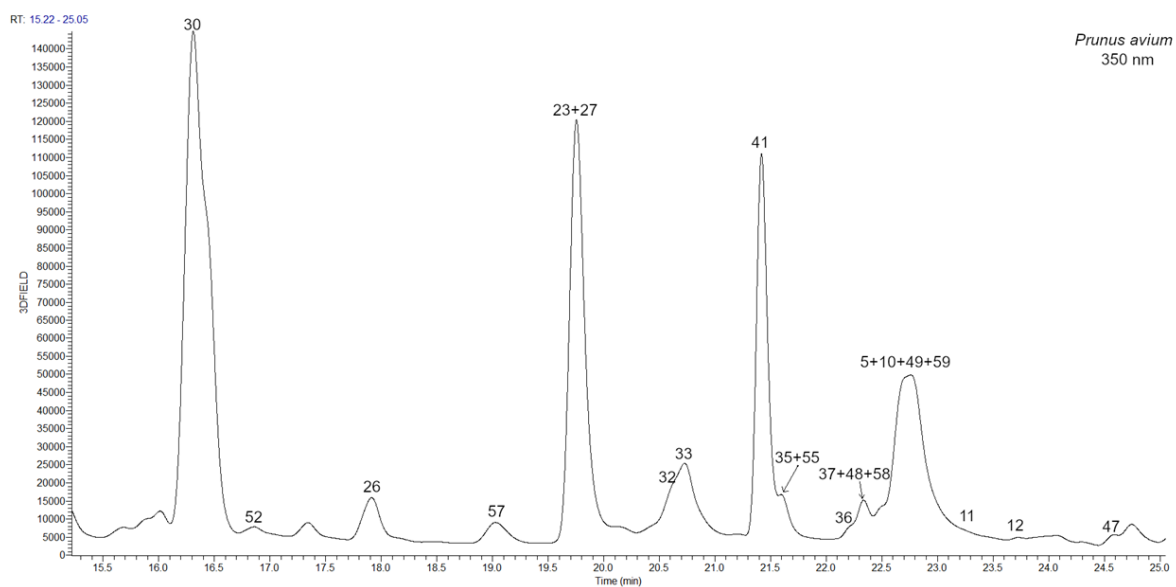

(B)

RT: 3.84 - 22.12

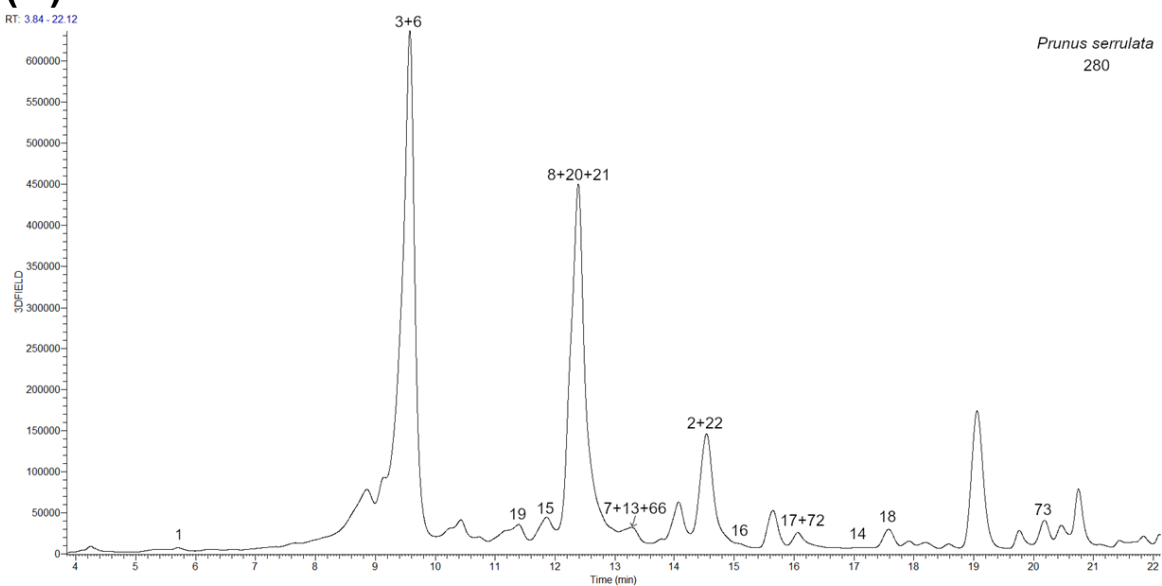

RT: 16.63 - 26.23

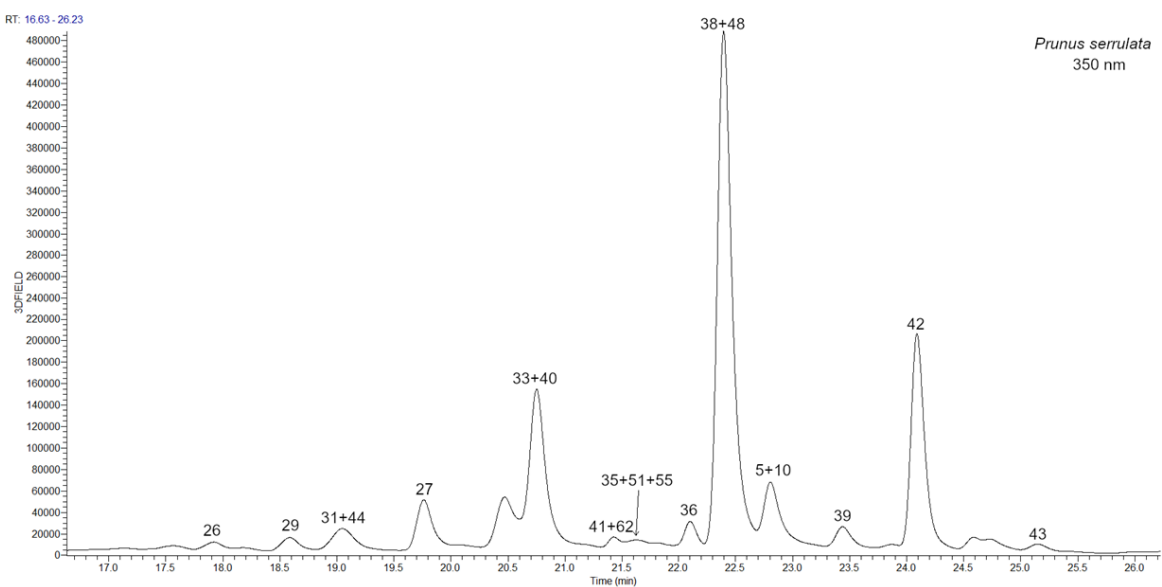

(C)

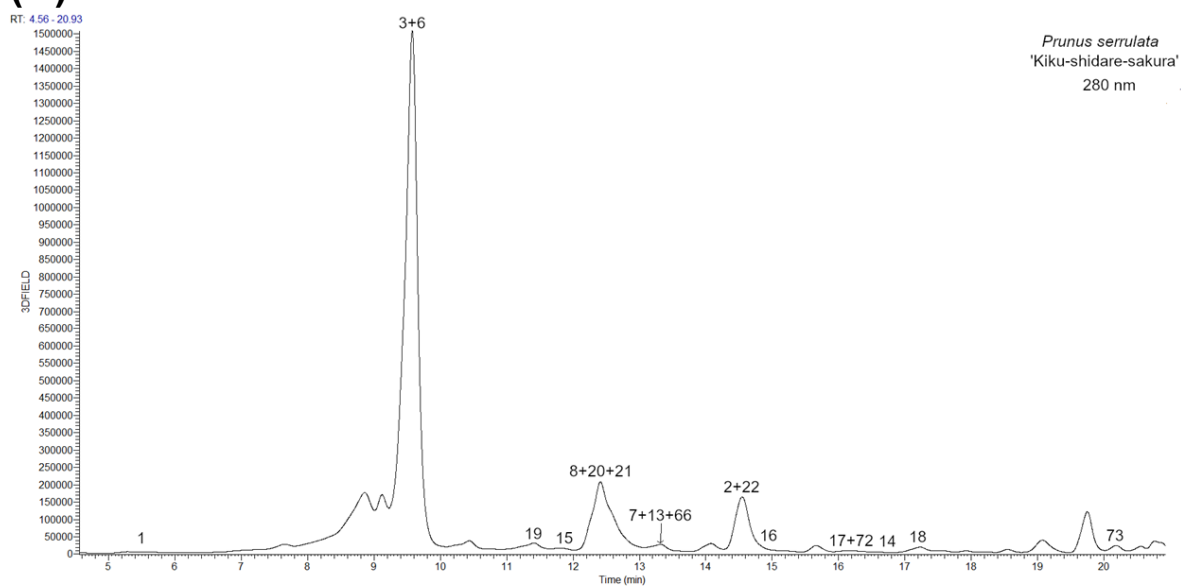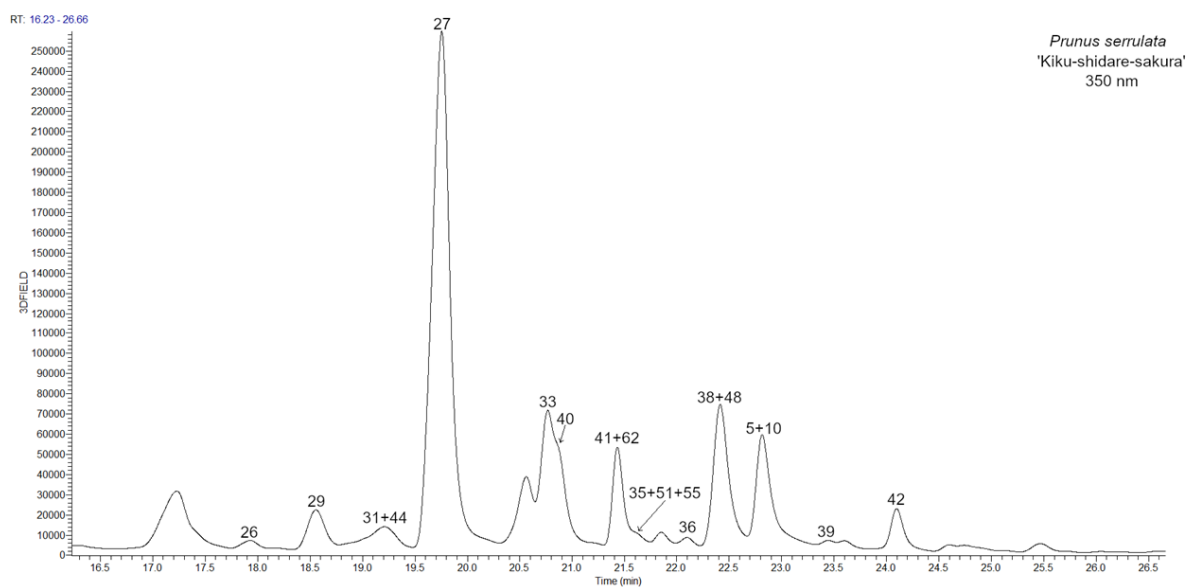

(D)

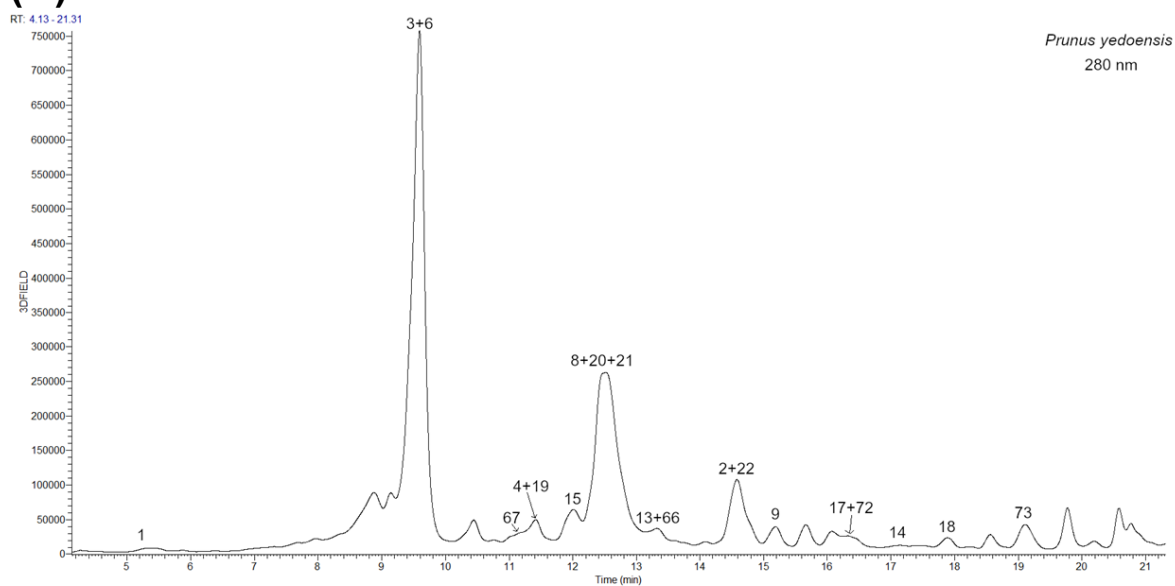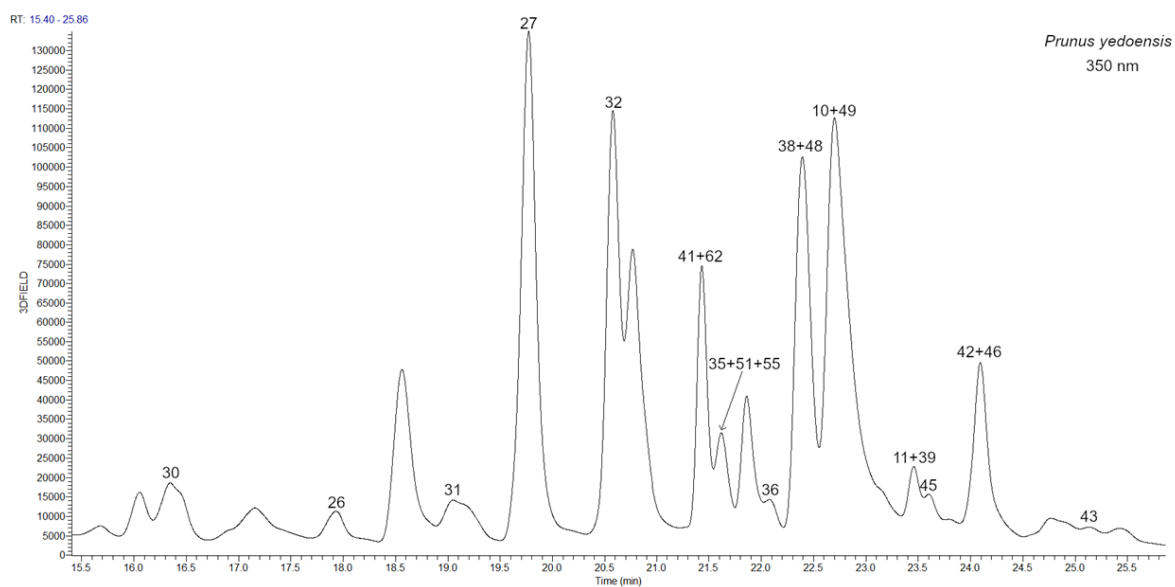

(E)

RT: 4.45 - 21.30

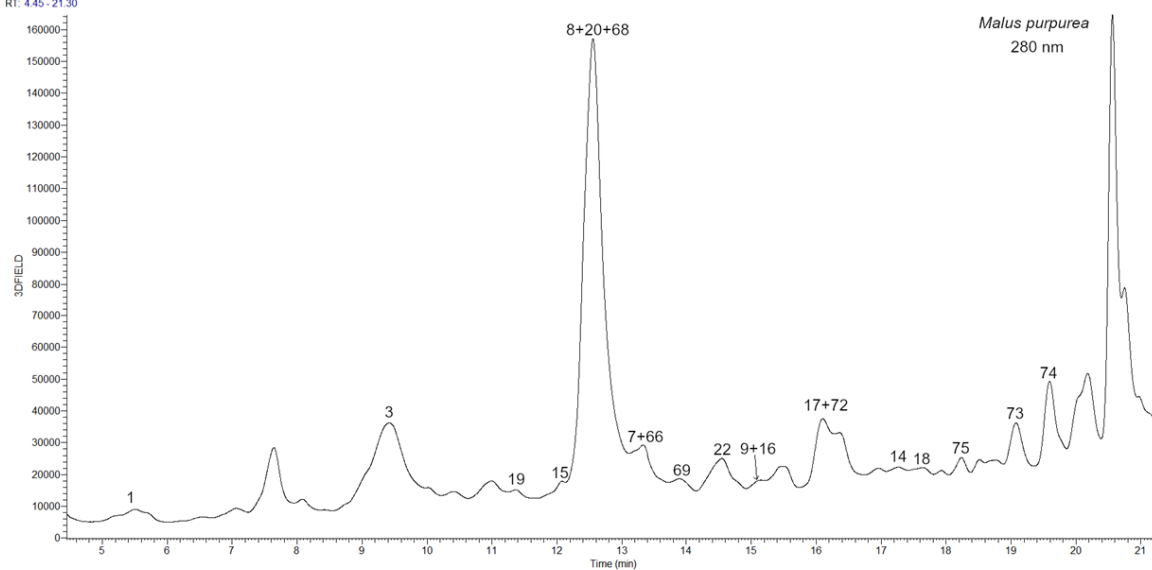

RT: 18.02 - 25.65

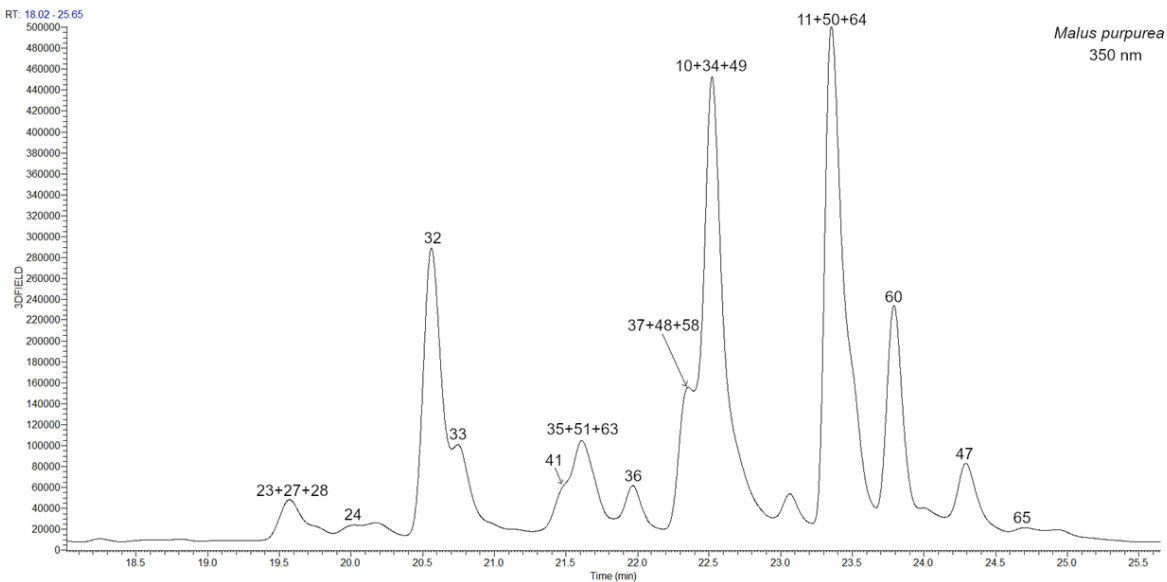

(F)

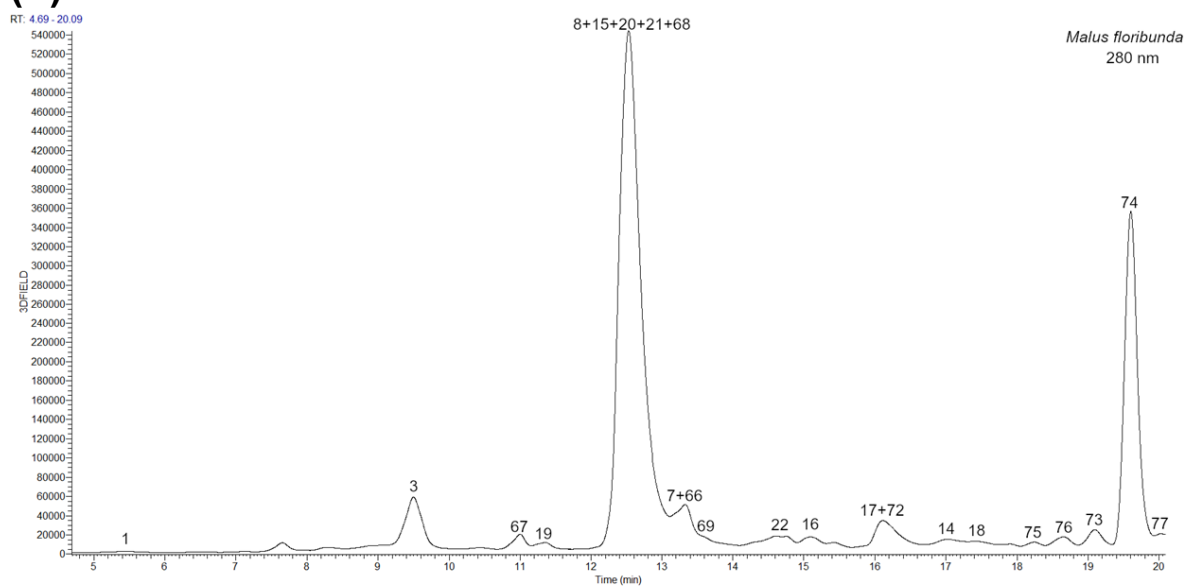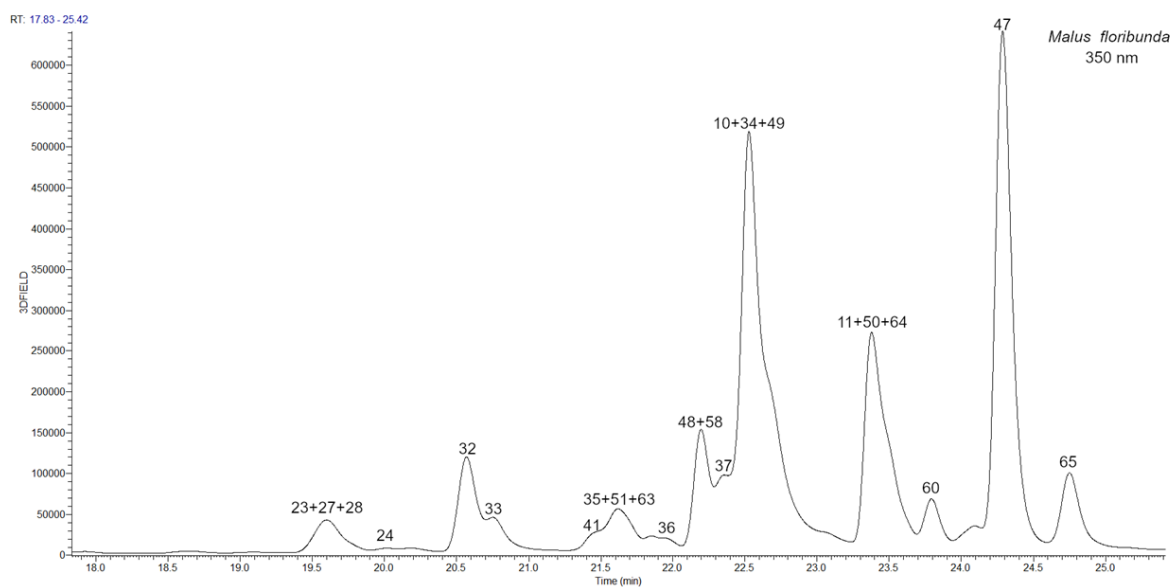

(G)

RT: 7.05 - 21.20

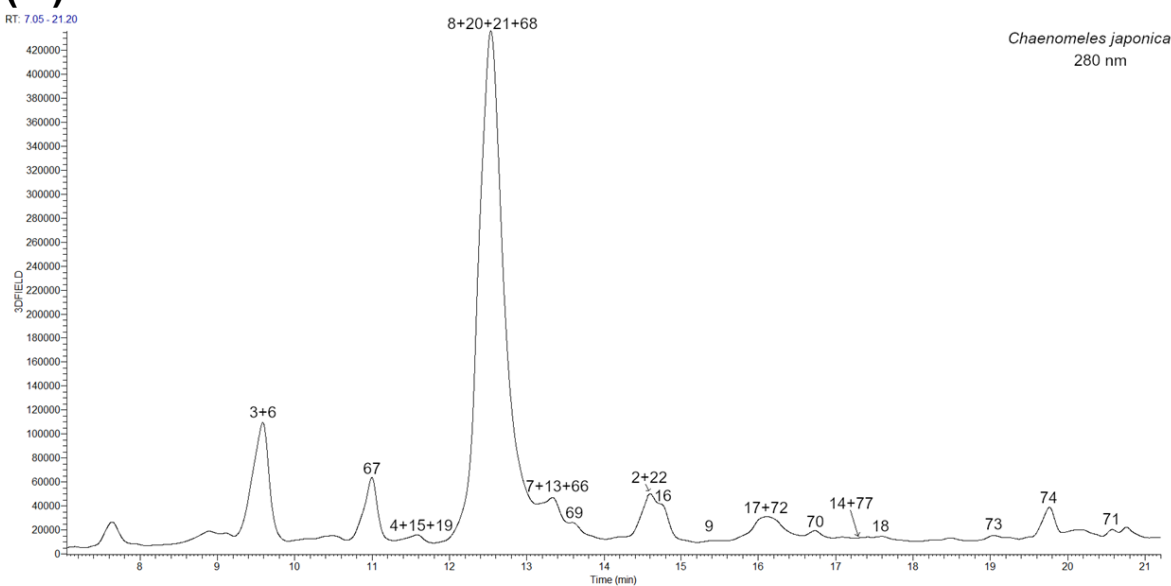

RT: 18.49 - 24.79

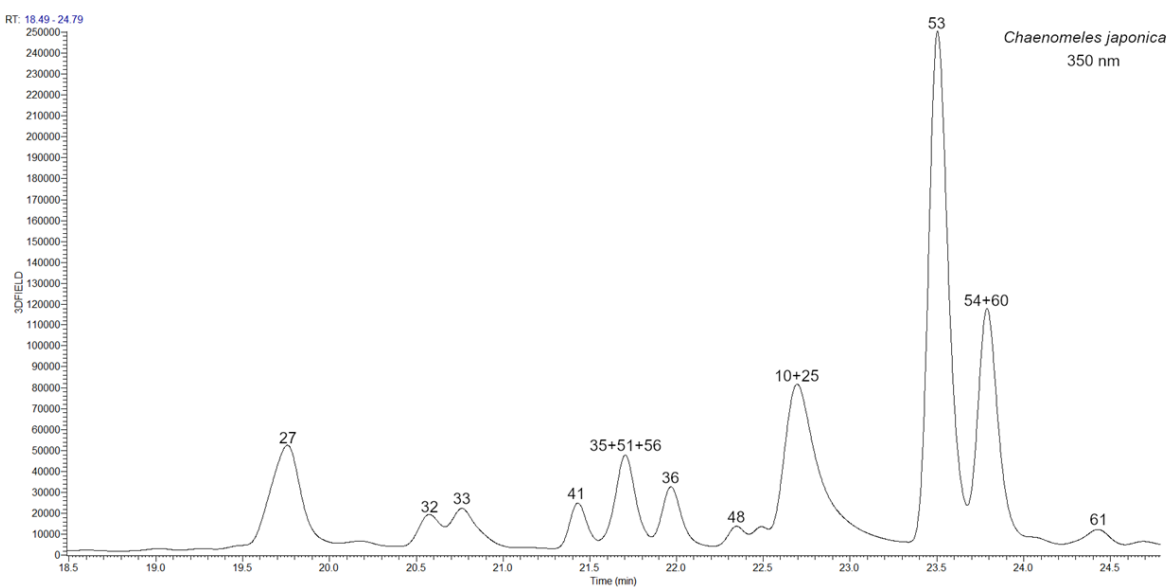

Supplement: Supplementary file 1 [file plants-11-00271-s001.zip › Figure S1.pdf]

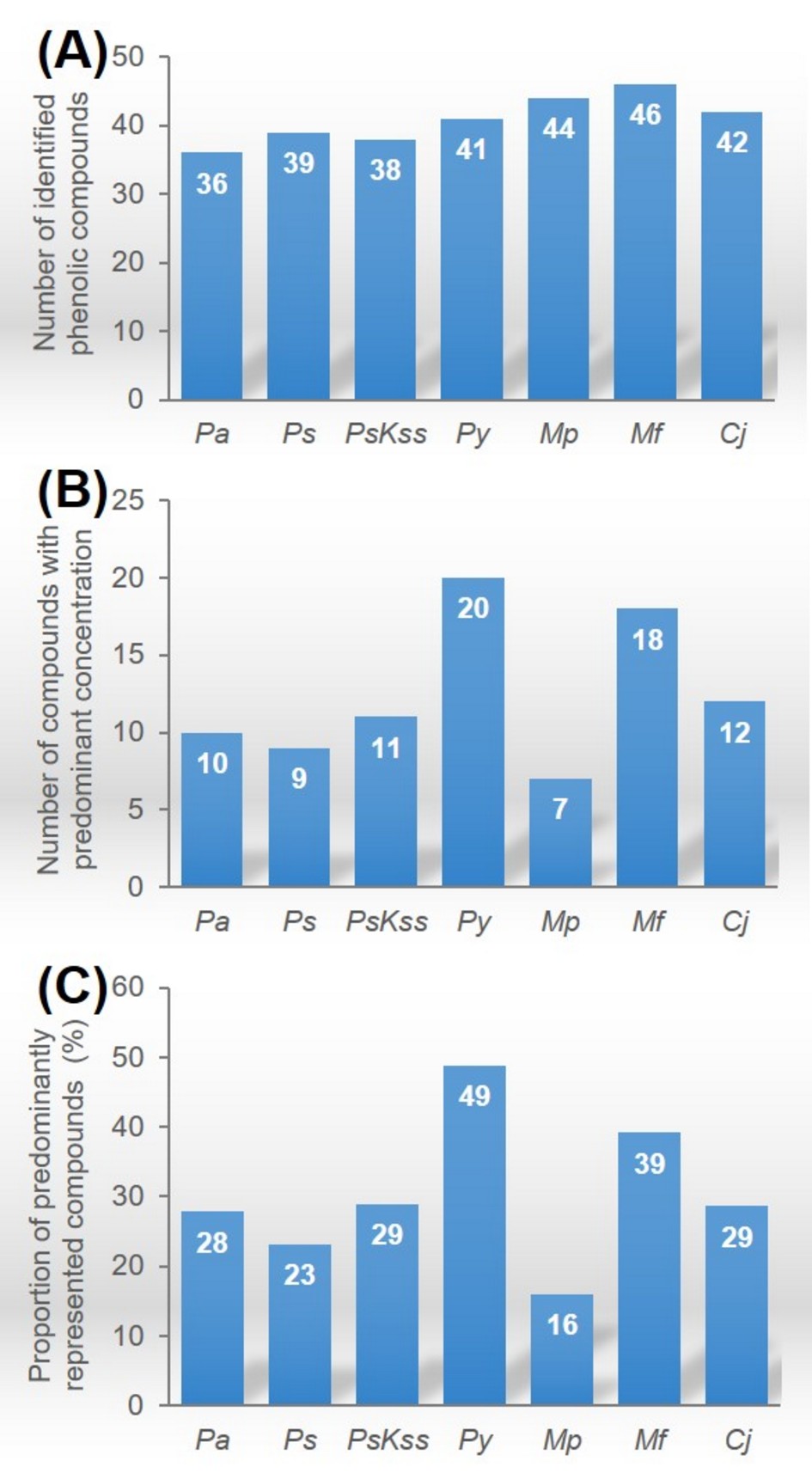

Supplement: Supplementary file 1 [file plants-11-00271-s001.zip › Figure S2.jpg]

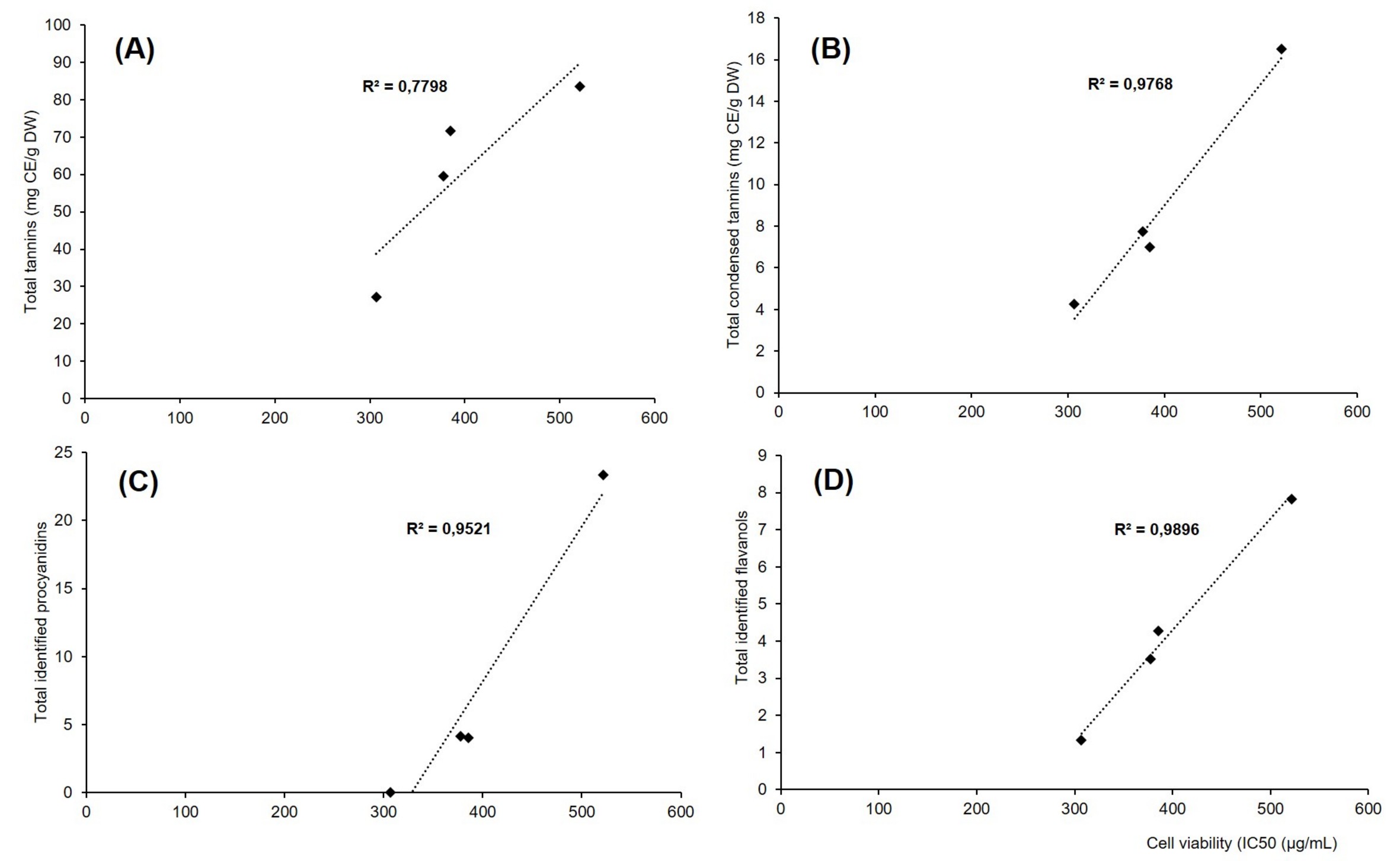

Supplement: Supplementary file 1 [file plants-11-00271-s001.zip › Figure S3.jpg]

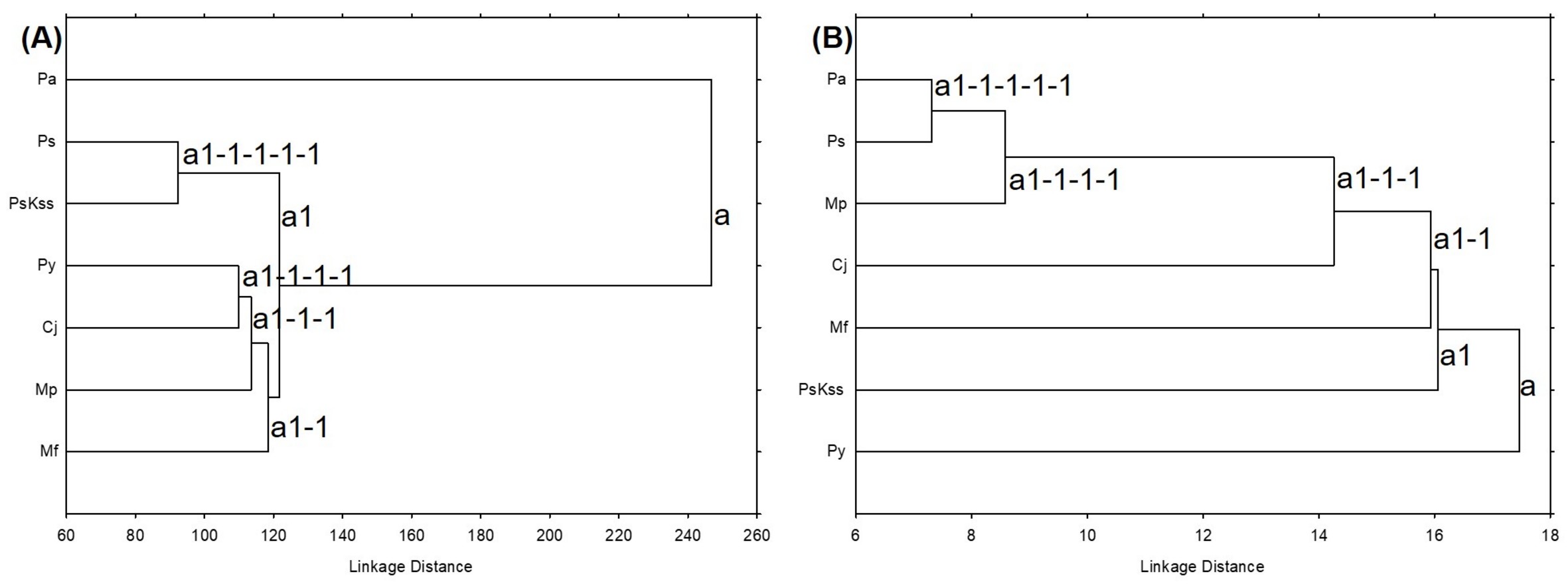

Supplement: Supplementary file 1 [file plants-11-00271-s001.zip › Figure S4.jpg]
